# Mechanism of Jianpixiaoke Recipe in Treating Type 2 Diabetes Based on Network Pharmacology and Molecular Docking

**DOI:** 10.1155/ije/4160656

**Published:** 2025-12-26

**Authors:** Huan Li, Wenrong An, Chengcheng Huang, Guowei Fang, Yanqin Huang

**Affiliations:** ^1^ Department of Cardiovascular, Yanqing Hospital of Beijing Chinese Medicine Hospital, Beijing, 102100, China; ^2^ Department of Endocrinology, The Second Affiliated Hospital of Shandong University of Traditional Chinese Medicine, Jinan, 250001, China, bucm.edu.cn; ^3^ Department of Endocrinology, Affiliated Hospital of Shandong University of Traditional Chinese Medicine, Jinan, 250014, China, sdutcm.edu.cn

**Keywords:** animal experiment, Jianpixiaoke recipe, molecular docking, network pharmacology, type 2 diabetes mellitus

## Abstract

**Objective:**

Using network pharmacology and molecular docking technology to analyze the potential mechanism of the Jianpixiaoke recipe (JPXK recipe) in the treatment of type 2 diabetes mellitus (T2DM).

**Method:**

This study searched and screened the active ingredients and corresponding T2DM‐related targets of different herbs in JPXK recipe from multiple databases. Venn was used to screen the intersection targets of JPXK recipe targets and T2DM targets, and the intersection targets were imported into the STRING database to construct a PPI network. In addition, clustering and topology analysis were also used to analyze PPI networks. At the same time, a composite target network was constructed using Cytoscape software. Then, we further carried out GO and KEGG enrichment analysis on the targets and carried out molecular docking verification on key chemical ingredients and hub targets. Finally, the effect of this prescription on T2DM was verified through animal experiments.

**Result:**

This study screened a total of 366 targets for the JPXK recipe, 1886 T2DM‐related targets, and 214 intersecting targets. The active ingredients of the JPXK recipe were quercetin, isorhamnetin, luteolin, berberine, kaempferol, etc. Potential hub targets were *ESR1*, *JUN*, *SRC*, *PIK3R1*, *FOS*, *MAPK1*, *AKT1*, *RELA*, and *MAPK3.* The main pathways of the JPXK recipe in treating T2DM included the MAPK signaling pathway, cAMP signaling pathway, tumor necrosis factor signaling pathway, Toll‐like receptor signaling pathway, etc. Molecular docking results showed that quercetin and luteolin combined best with the hub gene. *JUN*, *FOS*, *AKT1*, *RELA*, and *MAPK3* interacted well with these critical ingredients. Animal experiments verified that the JPXK recipe had a good hypoglycemic effect.

**Conclusion:**

This study revealed the active ingredients and potential molecular mechanism of the JPXK recipe in the treatment of T2DM, which provides valuable experience in future clinical trials.

## 1. Introduction

Type 2 diabetes mellitus (T2DM) was a chronic metabolic disease characterized by long‐term hyperglycemia. Its pathological factors were insulin resistance (IR), impaired islet function, etc. [1]. According to the data released by the International Diabetes Federation (IDF) in 2021, China had become the country with the most significant number of diabetes mellitus (DM). In the past 10 years, DM patients in China have increased from 90 million to 140 million. The number of DM patients in China is expected to reach 174.4 million in 2045 [2]. It can be seen that prevention and treatment of T2DM are particularly important.

Traditional Chinese Medicine (TCM) has been proven to be extremely effective in treating DM [3]. The prevention and treatment of DM with TCM have incomparable advantages, such as considering both specimens, overall regulation, stable curative effect, improving the quality of life, and is widely recognized in the clinical and experimental [4]. The research team adheres to Professor Yanqin Huang’s concept of “spleen deficiency causes diabetes” and founded the JPXK recipe which was from the classic famous “Xiaoke recipe” [5]. “JPXK recipe” consisted of *Astragalus membranaceus* (Huangqi, HQ), *Coptis chinensis Franch* (Huanglian, HL), *Radix trichosanthis* (Tianhuafen, THF), *Rehmannia glutinosa* (Shengdihuang, SDH), *Eupatorium fortunei Turcz* (Peilan, PL), and *Cyathula officinalis Kuan* (Chuanniuxi, CNX). All the TCMs in the JPXK recipe conformed to the pharmacological action and conventional dosage in the Pharmacopoeia of China. The recipe for invigorating the spleen and eliminating thirst has the effects of invigorating the spleen and qi, promoting blood circulation and removing blood stasis, clearing heat, and promoting fluid production, and was in line with the theory of Chinese medicine in treating DM. Confirmed by later clinical research that the application of specific dosages of JPXK recipe, as a safe and effective TCM recipe, could alleviate chronic inflammation by regulating the metabolites of gut microbiota, brain‐gut peptide, and adipocytokines, increasing the content of short chain fatty acids, thereby improving the rate of reaching the standard of blood pressure, blood sugar, and blood lipid in patients with metabolic syndrome, effectively improving the function of pancreatic islet β cells, promoting insulin secretion, and improving IR [6]. Experimental research confirmed that the application of specific dosages of JPXK recipe could regulate the intracellular transport pathway of cTAGE5/TANGO1, promote insulin synthesis and secretion, and improve Endoplasmic reticulum stress [7]. In order to further explore the hub targets and potential mechanisms of action of the JPXK recipe in the treatment of T2DM, we conducted this study.

Network pharmacology was a novel discipline based on system biology, molecular information science, pharmacology, and other aspects [8]. It had the characteristics of comprehensiveness and system integrity. It could analyze the multilevel network relationship of “molecular–target–pathway–disease” and clarifies the mechanism of Chinese herbal medicine remedies diseases from biological molecules and genes [9]. Molecular docking was also a new technology to predict the effectiveness of drug design according to the binding mode and affinity between molecules by simulating the binding between ligand molecules and receptor proteins [10]. With the unceasing improvement and development of network pharmacology and molecular docking, it has become a justifiable method for the research and development of TCM. And it is broadly used in the design of standard Chinese medicine compounds. The mechanism of TCM in the treatment of diseases will be sharper, and it equally provides a theoretical basis for the innovation of new drugs in the future.

**Figure 1:**
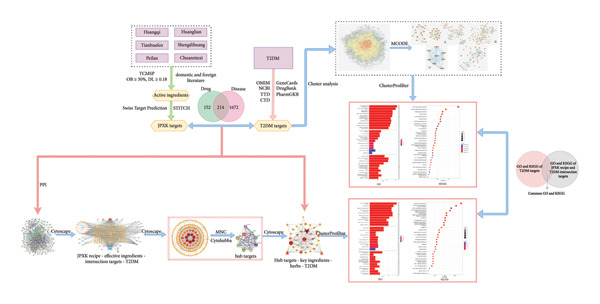
To explore the flowchart of the mechanism of the JPXK recipe in the treatment of T2DM. Abbreviation: Huangqi(HQ), *Astragalus membranaceus*; Huanglian(HL), *Coptis chinensis Franch*; Tianhuafen(THF), *Radix trichosanthis*; Shengdihuang(SDH), *Rehmannia glutinosa*; Peilan(PL), *Eupatorium fortunei Turcz*; Chuanniuxi(CNX), *Cyathula officinalis Kuan*.

This study explored the targets and pathways of the JPXK recipe in the treatment of T2DM through network pharmacology and molecular docking to clarify the mechanism of the JPXK recipe in the treatment of T2DM. The research technology is shown in Figure 1.

## 2. Materials and Methods

### 2.1. Effective Ingredients in the JPXK Recipe

Six herbs in the JPXK recipe were searched in the TCMSP database (https://old.tcmsp-e.com/tcmsp.php) to screen for effective active ingredients. But the effective active ingredients of SDH were not found in the database. The relevant chemical ingredients of SDH were supplemented by consulting the literature. The chemical ingredients of the JPXK recipe were imported into TCMSP. ADME includes the absorption, distribution, metabolism, and excretion of drugs. The higher the OB and DL values, the higher the absorption rate of the drug by the human body, which means the higher the available value of the drug. According to the pharmacokinetic ADME standard, all the ingredients were screened out for potentially effective ingredients according to the standards of oral bioavailability (OB) ≥ 30% and drug‐like (DL) ≥ 0.18. At the same time, the chemical ingredients that were effective but did not meet the screening conditions were searched on CNKI (https://www.cnki.net) and PubMed website (https://pubmed.ncbi.nlm.nih.gov/) to supplement them. Eventually, the effective ingredients of the JPXK recipe were obtained.

### 2.2. Targets Related to Effective Ingredients

Targets of the effective ingredients in the JPXK recipe were searched from the “Related Targets” of TCMSP. Considering the targets provided by TCMSP might be incomplete, the structural information of active ingredients was supplemented through PubChem database (https://pubchem.ncbi.nlm.nih.gov/), then the targets from the Swiss Target Prediction (https://www.swiss.target.prediction.ch/) and STITCH (https://stitch.embl.de/) were retrieved in this study, which could predict the targets of the active ingredients in the JPXK recipe. Swiss Target Prediction was a database for predicting molecular targets based on the similarity of 2D and 3D structures and the binding results with ligands [11]. In Swiss Target Prediction, the species was set to “Homo sapiens,” and the probability was no less than 0.6. STITCH was a database that integrates multiple literature and biological pathways to predict drug targets and their affinity [12]. In STITCH, the species was set to “Homo sapiens,” and the confidence score was ≥ 0.6. Eventually, the target in the JPXK recipe was imported into the UniProt database (https://www.uniprot.org/), limited the research species to “Homo,” converted the target into the corresponding gene name, and standardized it.

### 2.3. T2DM‐Related Targets

Take “type 2 diabetes mellitus” as the search keyword in GeneCards database (https://www.genecards.org/), TTD database (https://db.idrblab.net/ttd/), OMIM database (https://www.omim.org/), DrugBank database (https://go.drugbank.com/), NCBI database (https://www.ncbi.nlm.nih.gov/), CTD database (https://ctdbase.org/), and PharmGKB database (https://www.pharmgkb.org/) to select potential targets of T2DM. After merging 7 disease databases targets, delete the duplicate targets, and take the union set of the obtained targets as the disease candidate targets. The gene names corresponding to the targets were searched in the UniProt database, and the T2DM‐related targets were standardized.

### 2.4. Network of “JPXK Recipe–Effective Ingredients–Intersection Targets–T2DM”

Using VENNY 2.1 software (https://bioinfogp.cnb.csic.es/tools/venny/) to screen the intersection targets of JPXK recipe and T2DM targets, and draw a Venn diagram. The obtained JPXK recipe–T2DM intersection targets, the effective ingredients of corresponding Chinese medicine and disease were input into Cytoscape 3.9.0 software to construct the “JPXK recipe–effective ingredients–intersection targets–T2DM” network.

### 2.5. Networks of Protein–Protein Interaction (PPI)

#### 2.5.1. PPI Network of T2DM–JPXK Recipe and Hub Targets Network of T2DM–JPXK Recipe

The intersection targets protein of JPXK recipe–T2DM obtained in 2.4 was imported into the STRING database (https://string-db.org/) to construct a PPI network. The biological species were set as “Homo sapiens,” and the minimum confidence was 0.900. Simultaneously, the free points were hidden, and their parameters were selected as the default format. The drawn PPI network graphics and TSV files were downloaded and saved. Cytoscape 3.9.0 was used to construct the intersection target network of JPXK recipe–T2DM, and the Cytohubba plug‐in was used to filter hub genes. The top 10 genes generated by the maximum neighborhood component (MNC) method were hub genes. In each node in the interactive network, the degree value was used to measure the number of connections with other nodes, which often reflected the significance of nodes [13].

#### 2.5.2. PPI Network of T2DM and Cluster Analysis of T2DM

The PPI network of T2DM‐related targets was constructed by importing the T2DM‐related targets into the STRING database. The biological species was also set as “Homo sapiens,” and the minimum confidence was 0.900. Concurrently, the free points were hidden, and the parameters were selected as the default format. The PPI network graphics and TSV files were downloaded and saved. Cluster analysis can better filter out useful data, greatly improving the feasibility. The obtained PPI network was imported into Cytoscape 3.9.0, and the MCODE plug‐in was used for cluster analysis of the PPI network to select the same or similar nodes and protein complexes. When using the MCODE plug‐in in Cytoscape, the cut‐off value of node score = 0.2, *k* core = 2, maximum depth = 100, and degree cut‐off value (minimum score threshold) = 2 were set as the filtering conditions, and took the targets of the first five clusters for further GO and KEGG analysis.

### 2.6. GO and KEGG Enrichment Analysis

Using R 4.1.2 software, the symbols of the intersection targets of JPXK recipe–T2DM were converted into IDs, then Gene Ontology (GO) enrichment analysis and Kyoto Encyclopedia of Genes and Genomes (KEGG) were analyzed on them. The upshots were visualized, and the corresponding bar graph and bubble graph were plotted to analyze the possible mechanism of JPXK recipe treatment T2DM from the biological function and signaling pathway perspective. GO was a classification of gene functions, which is used to describe the properties of genes, including physical process (BP), cellular component (CC), and molecular function (MF). KEGG was a database based on gene and functional information, primarily used to analyze the function of genes and the chief metabolic pathways. GO and KEGG enrichment analysis were based on *p* < 0.05 to prepare the most prominent KEGG enrichment pathway.

### 2.7. Molecular Docking

Molecular docking between hub targets and key chemical ingredients was conducted. The ligand files of vital chemical ingredients were downloaded from the PubChem database and imported into Chem3D software for spatial structure transformation and energy optimization. The files were exported in MOL2 format and then saved in PDBQT format after processing by AutoDockTools software. The gene ID of the hub target was retrieved from the UniProt database, and the 3D structure of the protein receptor corresponding to the hub gene was obtained in the PDB database (https://www.rcsb.org/), and the corresponding PDB format file was downloaded. The macromolecular receptor file of the hub target was imported into AutoDockTools software for removing water molecules and hydrogenation, and the file was saved in PDBQT format. Ultimately, the hub targets and effective active ingredients were docked with AutoDock‐Vina software, and the binding energy was used as the docking evaluation index. For this research, 5 groups with the lowest binding energy were filtered for data visualization by Pymol software.

## 3. Experimental Verification

### 3.1. Experimental Materials

#### 3.1.1. Experimental Animals

Thirty‐six healthy SPF male Wistar rats, 4 weeks old, body weight (130 ± 10) g, purchased from Beijing Weitong Lihua Laboratory Animal Technology Limited Company, certificate number: SCXK (Beijing) 2016‐0006, raised in Shandong Animal Experiment Center, Affiliated Hospital of Shandong University of TCM. The ambient temperature was 18∼26°C, and the humidity was 60%∼70%. Keep breathing smoothly and had a circadian rhythm. The experiment passed the ethics review of the Affiliated Hospital of Shandong University of TCM, review number: AWE‐2019‐001.

#### 3.1.2. Experimental Drugs and Reagents

JPXK recipe (30 g of HQ, 9 g of HL, 12 g of THF, 12 g of SDH, 12 g of PL, 9 g of CNX, TCM granules, Jiangyin Tianjiang Pharmaceutical Limited Company).

Streptozotocin (STZ) (batch number: #18883‐66‐4), Sigma Company, USA; Rat Insulin ELISA Kit (batch number: #CSB‐E05070r), Rat Glucagon ELISA Reagent Box (batch number: #CSB‐E12800r); sodium citrate (batch number: #2018010203), Tianjin Zhiyuan Chemical Reagent limited company; citric acid (batch number: #2018‐09‐16), Tianjin Dingshengxin Chemical Limited Company.

#### 3.1.3. Experimental Instrument

Wenhao blood glucose meter and supporting blood glucose test strips (Johnson & Johnson (China) Medical Equipment Limited Company); Leica Immunofluorescence Microscope DM2500 (Leica, Germany).

### 3.2. Modeling and Drug Delivery

Twelve rats were randomly selected as a control group and fed with common feed throughout the whole process. The model group was fed with SPF grade high‐sugar and high‐fat diet for 8 weeks [14]. Then, the model group was fasted for 12 h without water and received a one‐time intraperitoneal injection of STZ (concentration 35 mg/kg), and the control group was intraperitoneally injected with an equal volume of citric acid buffer (0.1 mmol/L, pH = 4.3). After 72 h, blood was collected from the tail vein to measure the fasting blood glucose (FBG) of the rats in the model group. The FBG values of the rats in the model group and JPXK recipe group were higher than those in the control group and higher than 16.7 mmol/L (*p* < 0.05), indicating that the modeling was successful [14]. The 24 successfully modeled rats were randomly divided into a model group and a JPXK recipe group, with 12 rats in each group.

Refer to the equivalent dose conversion formula for adults (60 kg) and animals: DB = DA × KA/KB (DA represents the known dose in humans, DB represents the unknown dose in rats, KA and KB represent human and the body shape coefficient of rats, respectively), JPXK recipe group was given Chinese medicine decoction 3.125 g/kg by gavage. The control group and model group were given 3.125 g/kg 0.9% sodium chloride solution by gavage.

### 3.3. Pancreatic Tissue Collection and Sample Processing Assays

At the end of the experiment, the rats were dehydrated for 2 h, fasted for 12 h, then weighed, anesthetized by intraperitoneal injection of 2% pentobarbital sodium (0.2 mL/100 g), and dissected. 5 mL of blood was collected from the abdominal aorta, centrifuged at 4°C at 3000 r/min for 10 min, the upper serum was taken and placed in a refrigerator at −20°C. The pancreas specimen of appropriate size was taken from each group and fixed in 4% formaldehyde solution for immunofluorescence.

### 3.4. Determination of FBG, Fasting Serum Insulin (FINS), Serum Glucagon (GC), and IR Index (HOMA‐IR) in Rats

The Wenhao blood glucose meter was used to measure the changes in FBG in the treatment process; the FINS and GC levels of the rats were detected by the rat insulin Elisa kit, and HOMA‐IR = FBG × FINS/22.5 was calculated.

### 3.5. Detection by Immunofluorescence

The rat pancreatic tissue fixed in 4% formaldehyde was embedded in paraffin and sliced into pancreatic specimens with a thickness of 3 μm. After incubation and PBS treatment, the distribution of insulin and glucagon in pancreatic tissue was detected by a fluorescence microscope. The immunofluorescence used in this experiment was a positive control.

### 3.6. Statistical Analysis

Statistical analysis of the data was performed using SPSS 26.0 software. All measurement data were expressed as $\overset{¯}{x}$ ± *s*. Differences within groups were compared by independent samples *t*‐test, differences between groups were analyzed by one‐way analysis of variance (one‐way ANOVA), and *p* < 0.05 indicated that the differences were statistically significant. In this study, post hoc tests (*t*‐tests) were used to correct for significant ANOVA.

## 4. Results

### 4.1. Effective Chemical Ingredients of JPXK Recipe

Based on database search and literature search, the chemical ingredients were screened by OB ≥ 30% and DL ≥ 0.18: 20 ingredients of HQ, 14 ingredients of HL, 2 ingredients of THF, 7 ingredients of SDH, 12 ingredients of PL, and 4 ingredients of CNX. Ingredients with high gold content or vigorous activity reported in the literature that did not meet the above screening requirements were equally included. These ingredients included astragaloside I, astragaloside II, astragaloside III, astragaloside IV, gamma‐aminobutyric acid, gamma‐aminobutyric acid, catalpol, rehmannioside A, Stachyose, acteoside, HMF, gamma‐aminobutyric acid, ecdysteron, beta‐sitosterol. In the end, the active ingredients of the JPXK recipe were 25 active ingredients of HQ, 14 active ingredients of HL, 3 active ingredients of THF, 13 active ingredients of SDH, 12 active ingredients of PL, and 5 active ingredients of CNX. In this study, duplicate active ingredient were first deleted, and then active ingredient whose official targets could not be found in the UniProt database were deleted. Finally, 50 active ingredients were obtained (which are in Table 1).

**Table 1:** Effective chemical ingredients of JPXK recipe.

Drug	Molld	MolName	OB	DL
HQ	MOL000211	Mairin	55.38	0.78
HQ	MOL000239	Jaranol	50.83	0.29
HQ	MOL000296	Hederagenin	36.91	0.75
HQ	MOL000033	(3S,8S,9S,10R,13R,14S,17R)‐10,13‐dimethyl‐17‐[(2R,5S)‐5‐propan‐2‐yloctan‐2‐yl]‐2,3,4,7,8,9,11,12,14,15,16,17‐dodecahydro‐1H‐cyclopenta[a]phenanthren‐3‐ol	36.23	0.78
HQ	MOL000354	Isorhamnetin	49.6	0.31
HQ	MOL000371	3,9‐di‐O‐methylnissolin	53.74	0.48
HQ	MOL000374	5′‐hydroxyiso‐muronulatol‐2′,5′‐di‐O‐glucoside	41.72	0.69
HQ	MOL000378	7‐O‐methylisomucronulatol	74.69	0.30
HQ	MOL000379	9,10‐dimethoxypterocarpan‐3‐O‐beta‐D‐glucoside	36.74	0.92
HQ	MOL000380	(6aR,11aR)‐9,10‐dimethoxy‐6a,11a‐dihydro‐6H‐benzofurano[3,2‐c]chromen‐3‐ol	64.26	0.42
HQ	MOL000387	Bifendate	31.1	0.67
HQ	MOL000392	Formononetin	69.67	0.21
HQ	MOL000398	Isoflavanone	109.99	0.30
HQ	MOL000417	Calycosin	47.75	0.24
HQ	MOL000422	Kaempferol	41.88	0.24
HQ	MOL000433	FA	68.96	0.71
HQ	MOL000438	(3R)‐3‐(2‐hydroxy‐3,4‐dimethoxyphenyl)chroman‐7‐ol	67.67	0.26
HQ	MOL000439	Isomucronulatol‐7,2′‐di‐O‐glucosiole	49.28	0.62
HQ	MOL000442	1,7‐Dihydroxy‐3,9‐dimethoxy pterocarpene	39.05	0.48
HQ	MOL000098	Quercetin	46.43	0.28
HQ	MOL000401	astragalosideI	46.79	0.11
HQ	MOL000403	astragalosideII	46.06	0.13
HQ	MOL000405	astragalosideIII	31.83	0.10
HQ	MOL000407	astragalosideIV	22.50	0.15
HQ	MOL000388	Gamma‐aminobutyric acid	24.09	0.01
HL	MOL001454	Berberine	36.86	0.78
HL	MOL013352	Obacunone	43.29	0.77
HL	MOL002894	Berberrubine	35.74	0.73
HL	MOL002897	Epiberberine	43.09	0.78
HL	MOL002903	(R)‐Canadine	55.37	0.77
HL	MOL002904	Berlambine	36.68	0.82
HL	MOL002907	Corchoroside A_qt	104.95	0.78
HL	MOL000622	Magnograndiolide	63.71	0.19
HL	MOL000762	Palmidin A	35.36	0.65
HL	MOL000785	Palmatine	64.60	0.65
HL	MOL000098	Quercetin	46.43	0.28
HL	MOL001458	Coptisine	30.67	0.86
HL	MOL002668	Worenine	45.83	0.87
HL	MOL008647	Moupinamide	86.71	0.26
THF	MOL004355	Spinasterol	42.98	0.76
THF	MOL006756	Schottenol	37.42	0.75
THF	MOL000388	Gamma‐aminobutyric acid	24.09	0.01
SDH	MOL001525	Daucosterol	36.91	0.75
SDH	MOL002813	Aucubin	35.56	0.33
SDH	MOL000359	Sitosterol	36.91	0.75
SDH	MOL000449	Stigmasterol	43.83	0.76
SDH	MOL012254	Campesterol	37.58	0.71
SDH	MOL000519	Coniferin	31.11	0.32
SDH	MOL002819	Catalpol	5.07	0.44
SDH	MOL003730	Rehmannioside A	25.95	0.87
SDH	MOL000732	Stachyose	3.25	0.59
SDH	MOL003333	Acteoside	2.94	0.62
SDH	MOL000748	HMF	45.07	0.02
SDH	MOL000388	Gamma‐aminobutyric acid	24.09	0.01
SDH	MOL000358	Beta‐sitosterol	36.91	0.75
PL	MOL000006	Luteolin	36.16	0.25
PL	MOL000359	Sitosterol	36.91	0.75
PL	MOL000363	Amyrin palmitate	32.68	0.30
PL	MOL000449	Stigmasterol	43.83	0.76
PL	MOL000584	7‐acetoxy‐8‐hydroxy‐9‐isobutyryloxythymol	33.39	0.18
PL	MOL000588	9‐acetoxy‐8,10‐epoxy‐6‐hydroxythymol 3‐O‐angelate	61.44	0.21
PL	MOL000592	Dammaradienyl acetate	46.52	0.82
PL	MOL000595	Eupatoriopicrin	76.78	0.36
PL	MOL000596	[(3S,4aR,6aR,6aR,6bR,8aR,12S,12aR,14aR,14bR)‐4,4,6a,6b,8a,12,14b‐heptamethyl‐11‐methylene‐1,2,3,4a,5,6,6a,7,8,9,10,12,12a,13,14,14a‐hexadecahydropicen‐3‐yl] acetate	43.08	0.74
PL	MOL000604	Eupaformosanin	50.20	0.52
PL	MOL000605	Taraxasteryl palmitate	33.84	0.31
PL	MOL000358	Beta‐sitosterol	36.91	0.75
CNX	MOL012286	Betavulgarin	68.75	0.39
CNX	MOL012298	Rubrosterone	32.69	0.47
CNX	MOL000358	Beta‐sitosterol	36.91	0.75
CNX	MOL000098	Quercetin	46.43	0.28
CNX	MOL002212	Ecdysterone	5.30	0.82

Abbreviations: CNX, *Cyathula o%cinalis Kuan;* HL, *Coptis chinensis Franch;* HQ, *Astragalus membranaceus;* PL, *Eupatorium fortunei Turcz;* SDH, *Rehmannia glutinosa;* THF, *Radix trichosanthis*.

### 4.2. Prediction of Potential Targets of Effective Ingredients of JPXK Recipe

The “Related Targets” module was selected in the TCMSP database to obtain the corresponding targets of the effective ingredients of the JPXK recipe. A total of 366 gene targets of the JPXK recipe were obtained after merging and deduplication (as shown in Table 2).

**Table 2:** Effective ingredients and related targets of JPXK recipe.

Traditional Chinese Medicine	Effective ingredient	Target	Traditional Chinese Medicine	Effective ingredient	Target
HQ	19	605	SDH	10	169
HL	12	363	PL	7	195
THF	3	32	CNX	5	277

Abbreviations: CNX, *Cyathula o%cinalis Kuan;* HL, *Coptis chinensis Franch;* HQ, *Astragalus membranaceus;* PL, *Eupatorium fortunei Turcz;* SDH, *Rehmannia glutinosa;* THF, *Radix trichosanthis*.

### 4.3. Targets of T2DM

Taking “type 2 diabetes mellitus” as the keyword, 1039 targets in GeneCards database, 184 targets in the OMIM database, 83 targets in the TTD database, 157 targets in the DrugBank database, 957 targets in the NCBI database, 235 targets in CTD database, and 24 targets in PharmGKB database were retrieved in the disease database. The obtained T2DM‐related targets were combined, duplicate targets were eliminated, and 1886 disease‐related action targets was obtained (as shown in Figure 2(a)). By using VENNY 2.1 software, 366 JPXK recipe targets and 1886 T2DM‐related disease targets were crossed, and a Venn plot was drawn to obtain 214 intersection targets (as shown in Figure 2(b)).

Figure 2(a): Targets Venn map of T2DM‐related disease databases. (b): Intersection targets of JPXK recipe and T2DM. Abbreviation: drug represented JPXK recipe, disease represented T2DM.

**Figure:**
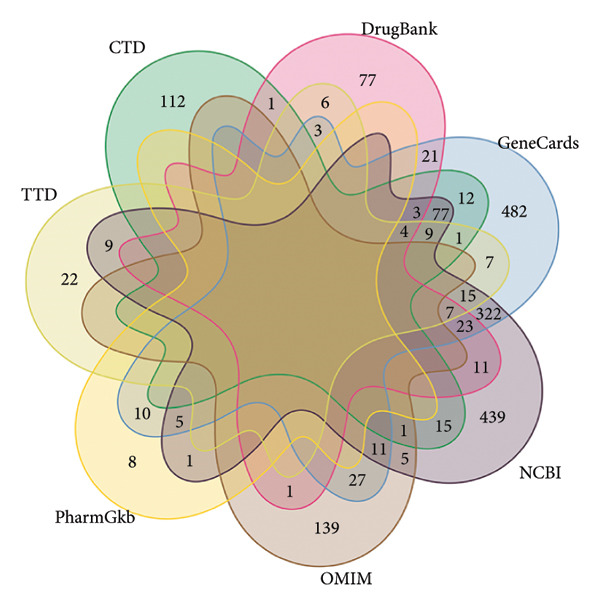
(a)

**Figure:**
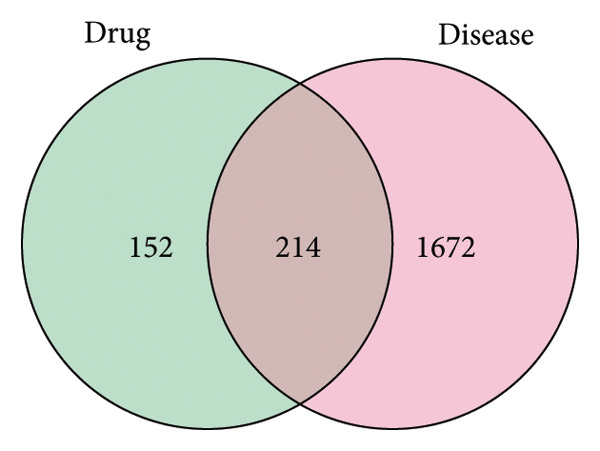
(b)

### 4.4. Network of “JPXK Recipe–Effective Ingredients–Intersection Targets–T2DM”

**Figure 3:**
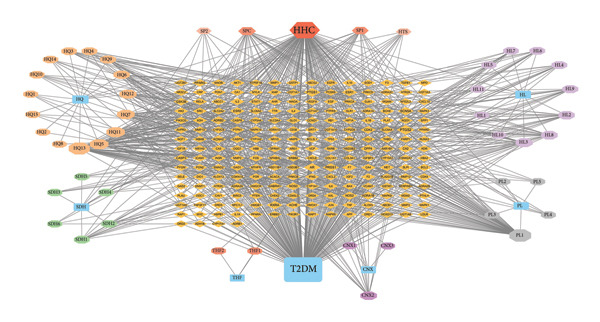
Network construction of “JPXK recipe–effective ingredients–intersection targets–T2DM”. Orange diamond represents the intersection targets, and the area of the node represents its degree value, indicating the importance of the node. Abbreviation: HQ, *Astragalus membranaceus*; HL, *Coptis chinensis Franch*; THF, *Radix trichosanthis*; SDH, *Rehmannia glutinosa*; PL, *Eupatorium fortunei Turcz*; CNX, *Cyathula officinalis Kuan*. HHC, the common components of HQ, HL, and CNX; SPC, the common components of SDH, PL, and CNX; HTS, the common components of HQ, THF, and SDH; SP1 and SP2, the common components of SDH and PL. HQ included HQ1, HQ2, HQ3, HQ4, HQ5, HQ6, HQ7, HQ8, HQ9, HQ10, HQ11, HQ12, HQ13, HQ14, HQ15, HHC, and HTS. HL included HL1, HL2, HL3, HL4, HL5, HL6, HL7, HL8, HL9, HL10, HL11, and HHC. THF included THF1, THF2, THF3, and HTS. SDH included SDH1, SDH2, SDH3, SDH4, SDH5, SDH6, SPC, HTS, SP1, and SP2. PL included PL1, PL2, PL3, PL4, PL5, SPC, SP1, and SP2. CNX included CNX1, CNX2, CNX3, SPC, and HHC. HQ1, MOL000033; HHC, MOL000098; HQ2, MOL000211; HQ3, MOL000239; HQ4, MOL000296; HQ5, MOL000354; HQ6, MOL000371; HQ7, MOL000378; HQ8, MOL000379; HQ9, MOL000380; HQ10, MOL000387; HTS, MOL000388; HQ11, MOL000392; HQ12, MOL000417; HQ13, MOL000422; HQ14, MOL000433; HQ15, MOL000442; HL1, MOL000622; HL2, MOL000785; HL3, MOL001454; HL4, MOL001458; HL5, MOL002668; HL6, MOL002894; HL7, MOL002897; HL8, MOL002903; HL9, MOL002904; HL10, MOL002907; HL11, MOL013352; THF1, MOL004355; THF2, MOL006756; SPC, MOL000358; SP1, MOL000449; SP2, MOL000359; SDH1, MOL000519; SDH2, MOL000748; SDH3, MOL002813; SDH4, MOL002819; SDH5, MOL003333; SDH6, MOL012254; PL1, MOL000006; PL2, MOL000584; PL3, MOL000588; PL4, MOL000595; PL5, MOL000604; CNX1, MOL002212; CNX2, MOL012286; CNX3, MOL012298.

The multidirectional network of “JPXK recipe–effective ingredients–intersection targets–T2DM” of JPXK recipe–T2DM was constructed by Cytoscape 3.9.0 software. The outcomes revealed that the network contained 268 nodes and 964 edges, including 47 effective ingredients and 214 intersection targets. The screening was carried out according to the degree values. Luteolin, kaempferol, isorhamnetin, formononetin, beta‐sitosterol, 7‐O‐methylisomucronulatol, berberine, calycosin, and stigmasterol were the top 10 chemicals with the degree values of 143, 65, 60, 37, 36, 29, 28, 25, 24, and 21, respectively. These chemical constituents might play a vital role in treating T2DM with the JPXK recipe (as shown in Figure 3).

### 4.5. Network Construction of PPI

#### 4.5.1. PPI Network of T2DM–JPXK Recipe and Network of “Hub Targets–Key Ingredients–Herbs–T2DM”

**Figure 4:**
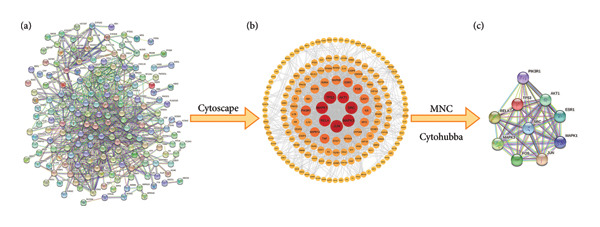
Identification of T2DM and JPXK recipe central genes. (a) PPI network of T2DM and JPXK recipe intersection targets. The network had 214 nodes and 848 edges. (b) Cytoscape map of T2DM and JPXK recipe intersection targets. (c) Hub targets of T2DM and JPXK recipe.

The intersection targets of 214 JPXK recipe–T2DM were imported into the String database for PPI analysis. The consequences exhibited that the network contained 214 nodes and 848 edges, with an average node degree of 7.93 (as shown in Figure 4 (a)). The downloaded PPI network corresponding TSV file was imported into Cytoscape 3.9.0 software, and the Cytohubba plug‐in in the Cytoscape platform was used to identify the hub gene (as shown in Figure 4(b)). The top 10 hub targets between JPXK recipe and T2DM were *ESR1*, *JUN*, *SRC*, *PIK3R1*, *FOS*, *MAPK1*, *AKT1*, *TP53*, *RELA*, and *MAPK3*. Among them, *SRC*, *PIK3R1*, *MAPK1*, *TP53*, *AKT1*, and *MAPK3* was also important target for T2DM, confirming the effectiveness of the JPXK recipe in treating T2DM. PPI network of 10 hub targets had 10 nodes and 45 edges; the average node degree was 9, and the *p*‐value was 0.000138 (as shown in Figure 4(c)).

**Figure 5:**
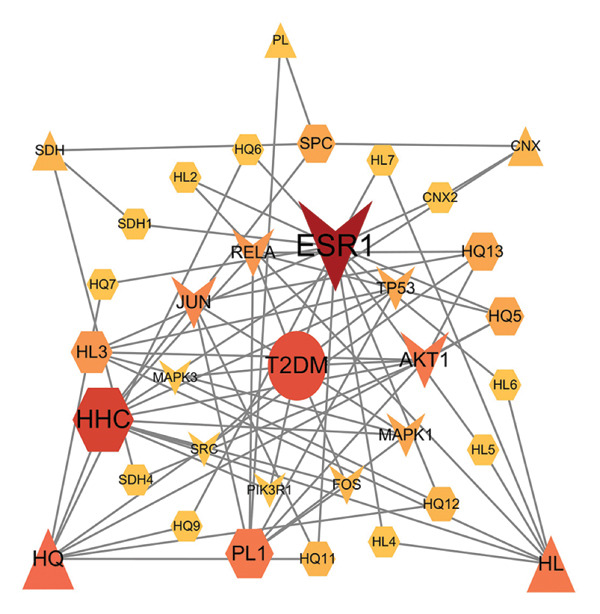
Network construction of “Hub targets–key ingredients–herbs–T2DM”. Circular nodes represent diseases, V‐shaped nodes represent hub targets, hexagonal nodes represent key ingredients related to hub targets, and triangular nodes represent herbs. The size and color of each node vary according to their degrees. Abbreviation: HQ, *Astragalus membranaceus*; HL, *Coptis chinensis Franch*; SDH, *Rehmannia glutinosa*; PL, *Eupatorium fortunei Turcz*; CNX, *Cyathula officinalis Kuan*. HHC, the common components of HQ, HL, and CNX; SPC, the common components of SDH, PL, and CNX. HHC, MOL000098; HQ5, MOL000354; HQ6, MOL000371; HQ7, MOL000378; HQ9, MOL000380; HQ11, MOL000392; HQ12, MOL000417; HQ13, MOL000422; HL2, MOL000785; HL3, MOL001454; HL4, MOL001458; HL5, MOL002668; HL6, MOL002894; HL7, MOL002897; SPC, MOL000358; SDH1, MOL000519; SDH4, MOL002819; PL1, MOL000006; CNX2, MOL012286.

A “Hub targets–key ingredients–herbs–T2DM” network was constructed based on the active ingredients related to hub targets. This network had 35 nodes and 73 edges (5 herbs, 19 key ingredients, 10 hub targets, and 1 disease). It could be seen that HQ had the most effective ingredients acting on the hub targets, suggesting that HQ had a good hypoglycemic effect. Quercetin, luteolin, berberine, kaempferol, and isorhamnetin were the critical components determined by the common targets–active ingredients network, which might be the material basis for treating T2DM with the JPXK recipe (as shown in Figure 5).

#### 4.5.2. Construction of PPI Network and Cluster Analysis of T2DM‐Related Targets

**Figure 6:**
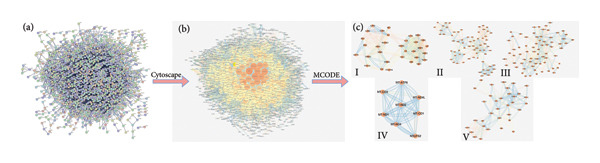
PPI network and cluster analysis of disease targets. (a) PPI network of T2DM targets. (b) Visualization of PPI network core targets. The red nodes in the middle represent the 10 targets with the maximum degree value. (c) The first five clustering diagrams from the PPI network of T2DM targets.

The String database analyzed the PPI network of 1886 T2DM‐related targets. The effects showed that the network contained 1716 nodes and 8385 edges, with an average node degree of 9.77 (as shown in Figure 6(a)).

The PPI network was introduced into Cytoscape 3.9.0, and the 10 targets with the most significant representation of red nodes in the middle (*SRC*, *PIK3R1*, *MAPK3*, *PIK3CA*, *MAPK1*, *EP300*, *TP53*, *STAT3*, *AKT1*, and *CTNNB1*) played a central role in the occurrence and progress of T2DM. The PPI network of T2DM‐related targets was clustered using the MCODE plug‐in in Cytoscape. That was why we got 48 clusters. We chose the top five clusters (As shown in Figure 6(b, c) and Table 3).

**Table 3:** Cluster analysis of PPI networks composed of T2DM‐related targets.

Cluster	Score	Nodes	Edges	Gene symbol
1	12.387	32	384	*IL2,IL2RA,IL2RB,IL2RG,VEGFA,IRS2,IL23A,OSM,MMP9,MMP1,LBP,IL10,FGF2,IL17A,POMC,IL6,LCN2,MET,TGFB1,TIMP1,ERBB2,MAP2K1,EGF,MMP3,MMP2,KRAS, ERBB3,PDGFRB,PIK3CB,IGF1R,NRAS,IRS1*
2	10.765	69	732	*CCL5,CCL3,IL18,IL1B,CCL2,CXCL10,PRKCZ,MYC,FGFR2,FGFR3,PIK3R1,PIK3CA,SHC1,JAK2,SOCS3,KIT,MAPK8,IL12A,IL1A,TNF,HRAS, KITLG,CXCL2,EGFR,SRC,SP1,HIF1A,MTOR, APOC3,NR1H3,PPARA, CETP,APOB, APOE,TSC1,LAMA2,CPT1A,MLST8,PRKAA2,DAG1,PRKAA1,LAMB2,LAMB3,PRKAG1,DMD,PRKAG2,PRKAB2,SMPD1,PRKAB1,LCAT, RAPSN,ITGA1,PAK1,LPA,LAMC2,LPL,TSC2,APOC2,FABP1,ACTA1,APOC1,APOA5,PRKAG3,JUN,INSR,SDC2,PRKCD, PTPN1,SMAD3*
3	8.464	70	584	*FOXO3,CXCL8,AGTR2,GNAS,RET,EPO,ADRB2,CCR5,HTR2C,ADRA1B,SOX2,RXRA,IL4R,RORA, BIRC5,FOS,BDNF,SOS1,INS,PIK3CG,FLT1,FLT4,IGF1,HDAC1,NKX2-5,SMAD5,BMP2,BMPR2,BMPR1A,GATA4,SMAD4,GRB2,NOTCH1,PTPN11,IL13,CAV1,IL6R,KDR,SMAD2,FOXO1,EP300,STK11,BRAF, CBLB,PRKCB,TEK,YWHAQ,FGF1,CD40,CD40LG,PTEN, MAPK1,ESR1,BMP7,BMP4,MAPK9,PTPN2,STAT5B,MAPK3,ARNT,KL,PLCG1,KLB,RPS6KB1,NR3C1,TLR4,FGF19,NTRK2,CDKN2A,SMARCA4*
4	7.429	8	52	*MT-ND4L,MT-ND4,MT-ND2,MT-ND1,MT-CO3,MT-CO1,MT-ATP6,NDUFS2*
5	7.273	34	240	*CDK4,TBP,RBPJ,LRP5,WNT5B,CCND2,WNT9A,WNT5A,GLI2,WNT3A,LRP6,WNT2B,WNT4,RELA,TYK2,CDK2,MDM2,CDKN2B,XRCC3,HDAC2,CDKN1B,TCF7L2,IFNB1,HDAC3,BRCA1,CTNNB1,RUNX2,EZH2,TP53,BRCA2,ATM,CDKN1A,CCND1,GSK3B*

### 4.6. GO and KEGG Analysis

#### 4.6.1. GO and KEGG Enrichment Analysis of JPXK Recipe–T2DM Intersection Targets

The intersection target of JPXK recipe–T2DM was converted By R 4.1.2 software, and the intersection target was performed by GO analysis. We got 3125 GO entries, including 2804 BP entries, 94 CC entries, and 227 MF entries. Since the *p*‐value represents enrichment and aboriginality, the first 10 items with the smallest *p*‐value in BP, CC, and MF were selected to make the GO bio function enrichment bar chart and bubble chart by R 4.1.2 software. The results presented that the BP of the intersection target genes of JPXK recipe and T2DM mainly concentrated in response to xenobiotic stimulation, wound healing, response to oxidative stress, response to nutrient levels, and response to lipopolysaccharide. CC was primarily focused on membrane raft, membrane microdomain, vesicle lumen, transcription regulator complex, and apical part of the cell. MF was mainly centered on DNA‐binding transcription factor binding, RNA polymerase II–specific DNA‐binding transcription factor binding, ubiquitin‐like protein ligase binding, cytokine receptor binding, and phosphatase binding. GO analysis results are shown in Figure 7(a). The enrichment of core targets in BP is shown in Figure 7(b), the enrichment of core targets in CC is shown in Figure 7(c), and the enrichment of core targets in MF is shown in Figure 7(d).

Figure 7GO analysis of intersection targets of JPXK recipe and T2DM.

**Figure:**
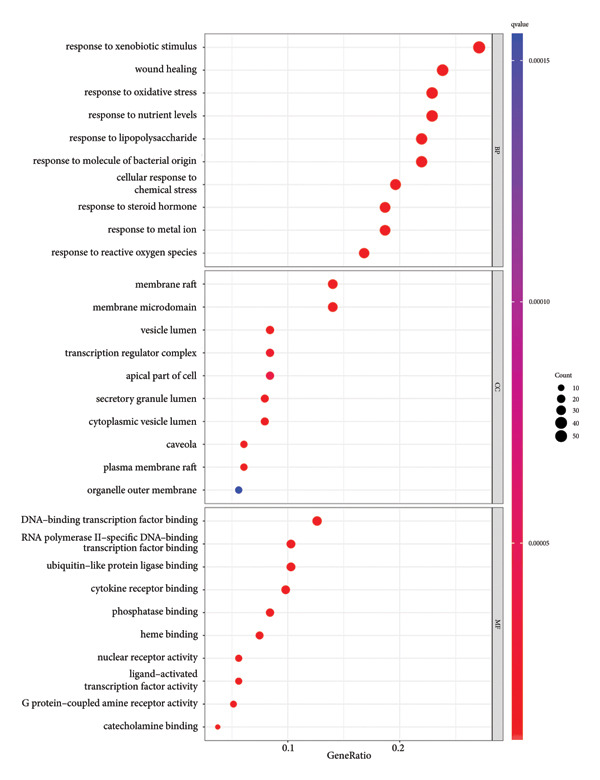
(a)

**Figure:**
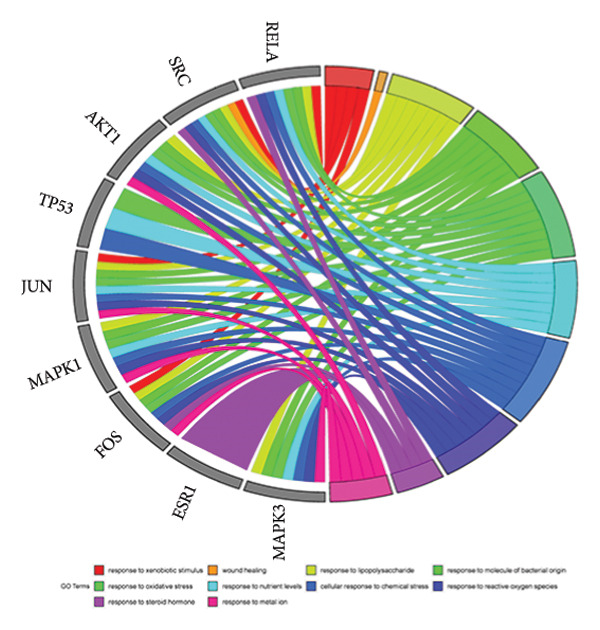
(b)

**Figure:**
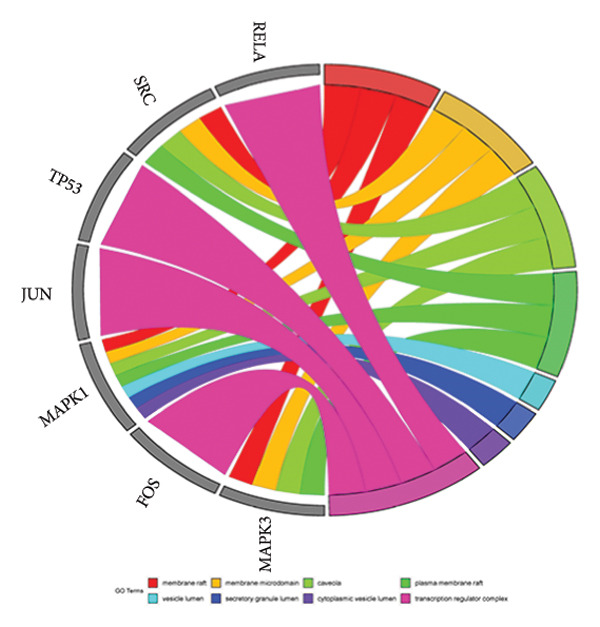
(c)

**Figure:**
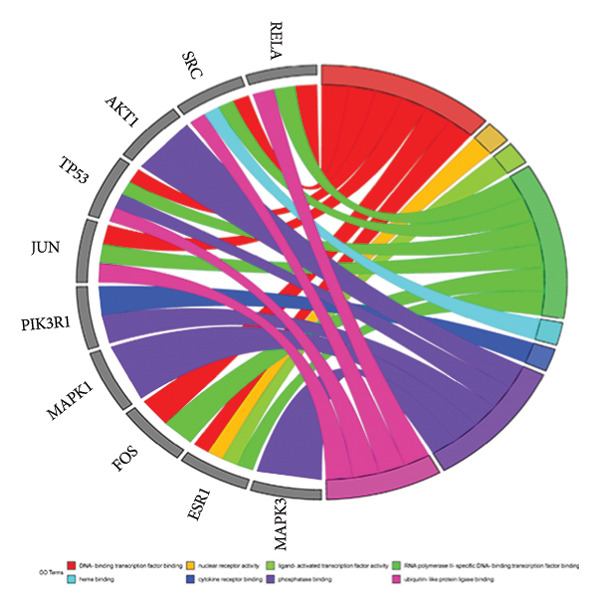
(d)

By using KEGG enrichment analysis, we obtained 187 KEGG signal pathways. The outcomes demonstrated that the signal pathway of the intersection targets between JPXK recipe and T2DM majorly touched upon lipid and atherosclerosis, the role of the AGE‐RAGE signaling pathway in diabetic complications, PI3K‐Akt signaling pathway, IL‐17 signaling pathway, fluid shear stress, and atherosclerosis, and endocrine resistance (As shown in Figure 8(a)). The enrichment of core targets in important pathways is shown in the figure below. The enrichment results of core targets in significant pathways are shown in Figure 8(b) . The results showed that the pathway most significantly enriched *PIK3R1*, *MAPK1*, *MAPK3*, and *AKT1* targets.

Figure 8(a) KEGG analysis of intersection targets of JPXK recipe and T2DM; (b) enrichment results of hub targets in significant pathways.

**Figure:**
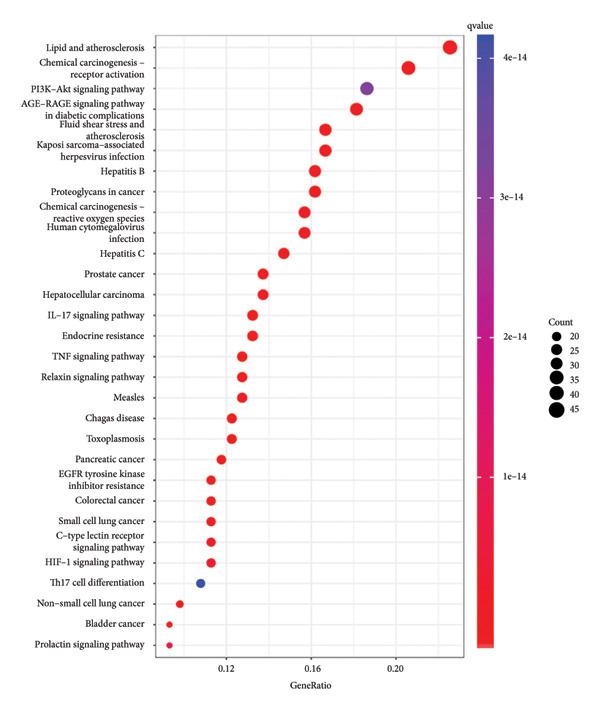
(a)

**Figure:**
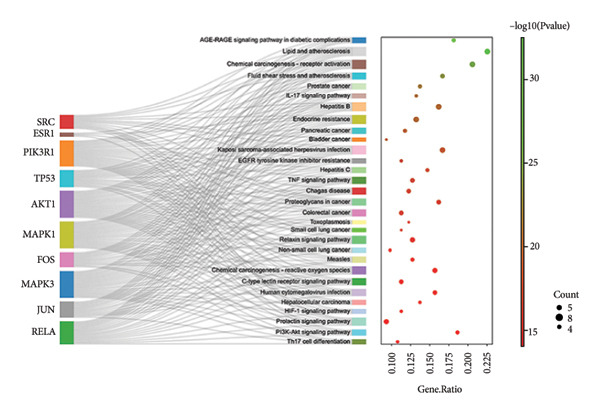
(b)

#### 4.6.2. GO and KEGG Analysis of T2DM‐Related Targets

GO and KEGG analyses were performed on the target points of five clusters obtained in Section 4.5.2 for T2DM disease. We ultimately received 3138 BP entries, 224 MF entries, 96 CC entries, and 182 KEGG pathways. The top 10 meaningful GO and the top 30 meaningful KEGG pathways in BP, MF, and CC are shown in the figure, respectively.

GO analysis revealed that the BP of T2DM was mainly affected by the positive regulation of kinase activity, positive regulation of *MAPK* cascade, peptidyl‐tyrosine phosphorylation, and peptidyl‐tyrosine modification, positive regulation of protein kinase activity, and regulation of protein serine/threonine kinase activity. The CC related to T2DM could be found in the transcription regulator complex, membrane raft, membrane microdomain, transferase complex, transferring phosphorus‐containing groups and protein kinase complex activated. The MF of T2DM was related to cytokine activity, signaling receptor activator activity, receptor‐ligand activity, cytokine receptor binding, and transmembrane receptor protein kinase activity. GO analysis results are shown in Figure 9(a). The enrichment of core targets in BP is shown in Figure 9(b), the enrichment of core targets in CC is shown in Figure 9(c), and the enrichment of core targets in MF is shown in Figure 9(d).

Figure 9GO analysis of T2DM‐related targets.

**Figure:**
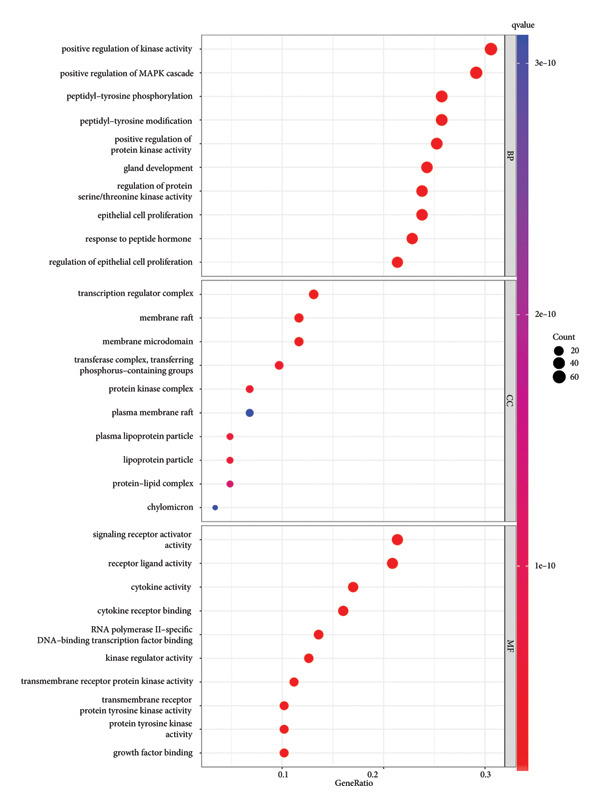
(a)

**Figure:**
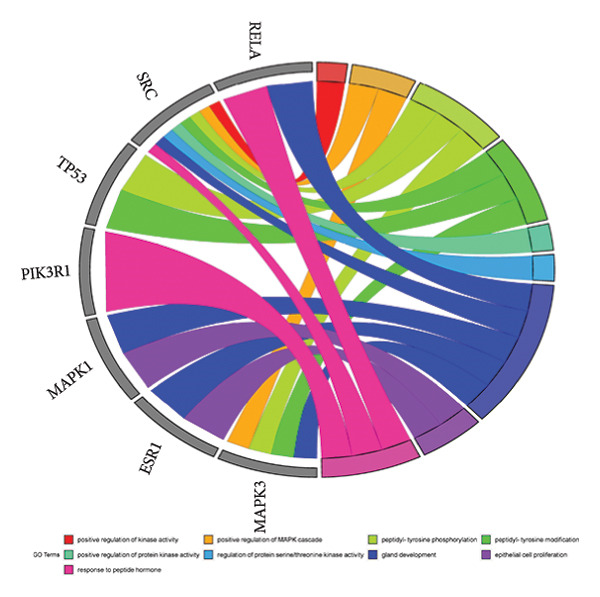
(b)

**Figure:**
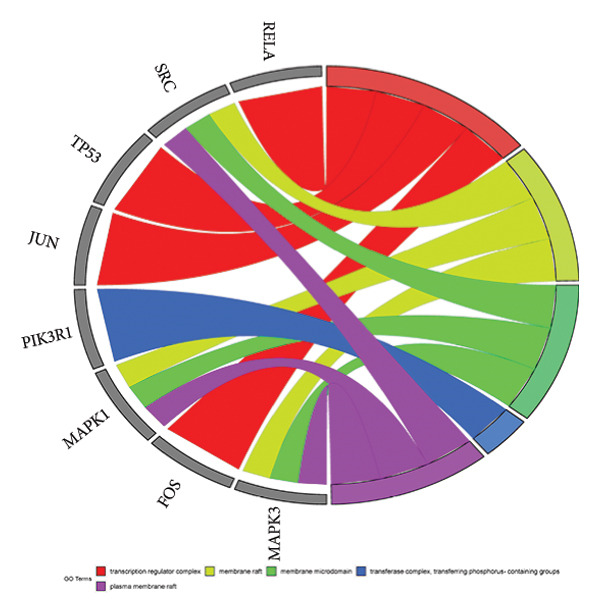
(c)

**Figure:**
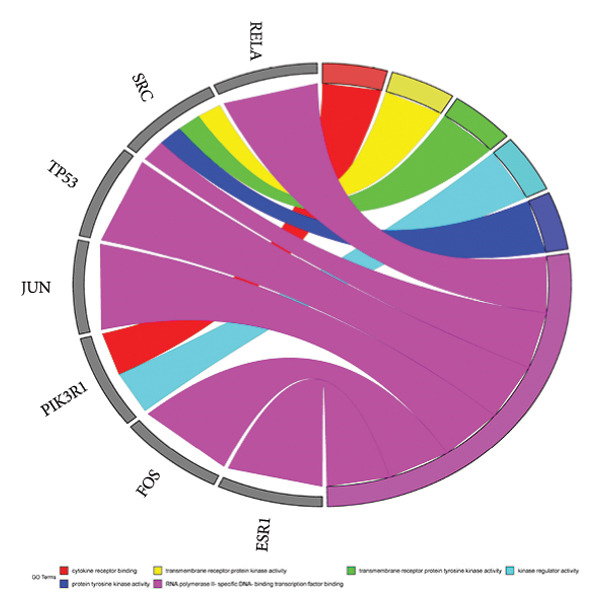
(d)

KEGG analysis displayed that the significant avenues of approach related to T2DM were PI3K‐Akt signaling pathway, FoxO signaling pathway, endocrine resistance, *MAPK* signaling pathway, mTOR signaling pathway, and AGE‐RAGE signaling pathway in diabetic complications (as shown in Figure 10(a)). The enrichment of core targets in important pathways is shown in Figure 10(b). The results showed that the pathway most significantly enriched in *PIK3R1, MAPK1*, and *MAPK3*.

Figure 10(a) KEGG analysis of T2DM‐related targets; (b) enrichment results of hub targets in significant pathways.

**Figure:**
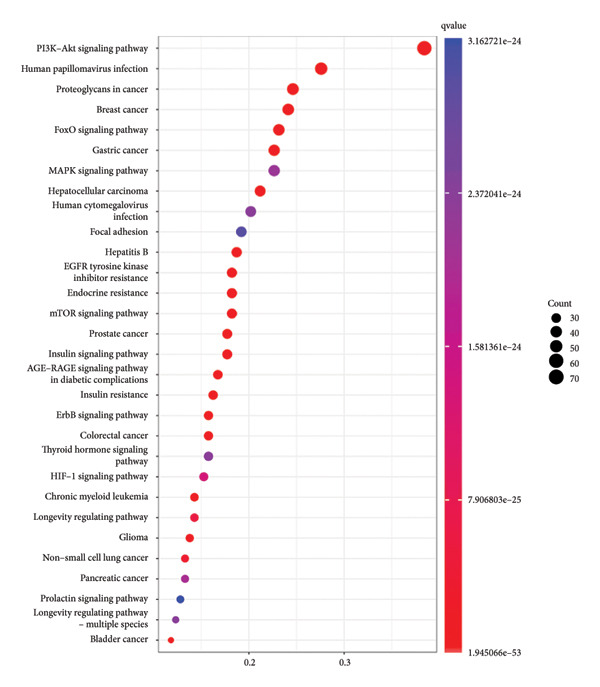
(a)

**Figure:**
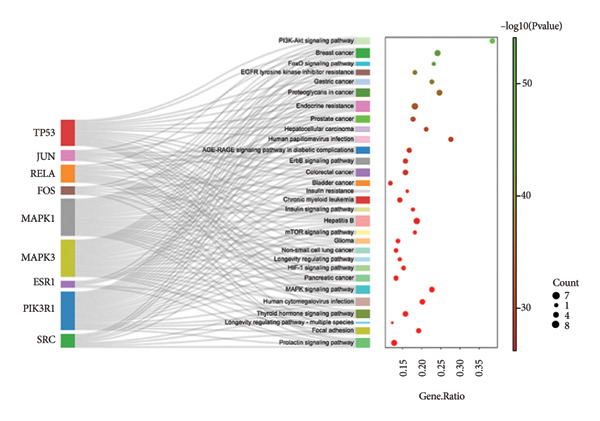
(b)

#### 4.6.3. Selection and Analysis of Crucial GO and KEGG of T2DM–JPXK Recipe

After comparing and analyzing the KEGG and GO functions of disease targets and common targets of JPXK recipe and T2DM, we found 2173 overlapping BPs, 112 overlapping MFs, 42 overlapping CCs, and 159 overlapping KEGG pathways. The hub genes selected by the MNC method might play a more meaningful role through the JPXK recipe in treating T2DM. Hence, we decided on GO with more than five hub targets and KEGG with more than 6 hub targets. This study obtained 16 critical BP, 4 vital CC, and 34 key KEGG pathways. Since no more than 5 hub targets were found in CC, only 1 CC containing more than 4 hub targets was selected, and chosen in the following analysis.

The key MF was mainly related to DNA binding transcriptional activation activity (GO:0001216), DNA binding transcriptional activation activity, RNA polymerase II specificity (GO:0001228), RNA polymerase II specificity, DNA binding transcription factor binding (GO:0061629), and DNA binding transcription factor binding (GO:0140297). The key CC were transcriptional regulators (GO:0005667). The critical BP was mainly involved in the reaction of lipopolysaccharide (GO:0032496), molecular response to bacterial sources (GO:0002237), cell response to biological stimulation (GO:0071216), oxidative stress (GO:0006979), reactive oxygen species (GO:0000302), nutritional response (GO:0031667), transmembrane receptor protein serine/threonine kinase signaling pathway (GO:0007178), etc. This suggests that the prevention of DM might be closely related to intestinal flora [15]. Studies had shown that intestinal flora was related to insulin release. Changes in the structure of intestinal flora will lead to changes in material metabolism in the body, damage to the intestinal wall, and infiltration of a large number of microorganisms into the blood and tissues, inducing inflammatory reactions and IR, resulting in disorders of blood glucose metabolism in T2DM patients [16, 17]. Some studies indicated that DM originated from “intestinal heat”, and the JPXK recipe was used to treat T2DM by the invigorating spleen and clearing heat, which was consistent with the network pharmacology mechanism in this study [18].

The clear KEGG enrichment pathway indicated that the JPXK recipe could play a synergistic role in the intervention of T2DM in protecting the function of islet β cells by regulating multiple signaling pathways such as immunity, hormones, and inflammatory reactions, anticancer, and anti‐infection. The prominent KEGG pathway is shown in Table 4.

**Table 4:** T2DM and its overlapping KEGG enrichment pathways with the JPXK recipe.

ID	Description	T2DM significant signal pathway			JPXK recipe ‐ T2DM significant signal pathway		
		p.adjust	Count	Number of hub genes	p.adjust	Count	Number of hub genes
hsa04010	MAPK signaling pathway	9.30E‐24	46	6	7.09E‐12	32	7
hsa04024	cAMP signaling pathway	4.11E‐06	19	6	7.84E‐05	17	7
hsa04668	TNF signaling pathway	8.86E‐14	22	6	2.95E‐17	26	7
hsa04919	Thyroid hormone signaling pathway	1.01E‐23	32	6	7.03E‐11	20	7
hsa04915	Estrogen signaling pathway	8.39E‐13	23	7	1.58E‐07	17	8
hsa04917	Prolactin signaling pathway	1.37E‐23	26	7	3.37E‐14	19	8
hsa04926	Relaxin signaling pathway	1.99E‐17	27	7	8.82E‐16	26	8
hsa01522	Endocrine resistance	2.05E‐33	37	8	7.92E‐20	27	9
hsa04620	Toll‐like receptor signaling pathway	1.59E‐15	23	6	4.48E‐12	20	7
hsa04625	C‐type lectin receptor signaling pathway	1.11E‐17	25	6	5.86E‐15	23	7
hsa04660	T Cell receptor signaling pathway	1.11E‐17	25	6	5.99E‐13	21	7
hsa04662	B Cell receptor signaling pathway	2.85E‐10	16	6	2.07E‐06	12	7
hsa05418	Fluid shear stress and atherosclerosis	1.41E‐16	27	6	5.06E‐23	34	7
hsa05417	Lipid and atherosclerosis	1.32E‐21	38	8	1.06E‐28	46	9
hsa04722	Neurotrophin signaling pathway	2.71E‐17	26	6	1.88E‐08	17	7
hsa04210	Apoptosis	1.22E‐08	18	7	1.62E‐12	23	8
hsa04380	Osteoclast differentiation	6.86E‐10	19	6	2.58E‐11	21	7
hsa05205	Proteoglycans in cancer	4.23E‐35	50	6	7.09E‐17	33	7
hsa05235	PD‐L1 expression and PD‐1 checkpoint pathway in cancer	5.91E‐16	22	6	2.39E‐11	18	7
hsa05203	Viral carcinogenesis	7.87E‐16	31	7	2.90E‐05	17	7
hsa05208	Chemical carcinogenesis ‐ reactive oxygen species	6.66E‐14	30	7	5.36E‐15	32	8
hsa05207	Chemical carcinogenesis–receptor activation	4.02E‐17	33	8	9.79E‐25	42	9
hsa05210	Colorectal cancer	9.31E‐29	32	6	8.41E‐17	23	7
hsa05224	Breast cancer	2.38E‐41	49	7	7.12E‐12	23	8
hsa05130	Pathogenic *Escherichia coli* infection	4.97E‐05	16	6	2.69E‐07	20	6
hsa05131	Shigellosis	3.14E‐11	28	7	3.48E‐08	24	8
hsa05135	Yersinia infection	8.38E‐14	24	7	8.98E‐11	21	8
hsa05142	Chagas disease	6.91E‐18	25	6	3.18E‐17	25	7
hsa05171	Coronavirus disease ‐ COVID‐19	3.99E‐11	27	6	2.20E‐09	25	6
hsa05170	Human immunodeficiency virus 1 infection	9.28E‐10	24	6	4.38E‐08	22	7
hsa05163	Human cytomegalovirus infection	1.01E‐23	41	6	6.43E‐15	32	7
hsa05166	Human T‐cell leukemia virus 1 infection	4.57E‐22	39	7	1.10E‐12	29	8
hsa05161	Hepatitis B	5.32E‐26	38	8	7.76E‐20	33	9
hsa05167	Kaposi sarcoma–associated herpesvirus infection	3.35E‐22	37	8	1.85E‐18	34	9

By constructing the PPI network of T2DM and JPXK recipe, combined with Cytohubba identification of hub targets, GO and KEGG results were sorted and analyzed. The effective ingredients of the JPXK recipe in the treatment of T2DM were quercetin, luteolin, berberine, isorhamnetin, and kaempferol, and the essential targets were *ESR1*, *JUN*, *SRC*, *PIK3R1*, *FOS*, *MAPK1*, *AKT1*, *TP53*, *RELA*, and *MAPK3*.

### 4.7. Molecular Docking Results

**Figure 11:**
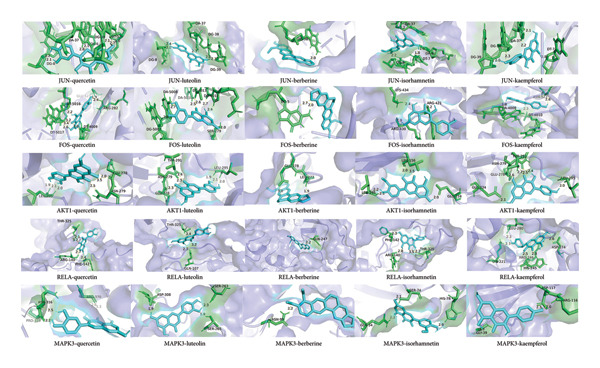
Molecular docking of key ingredients and hub targets.

Ten hub targets and 5 key chemical ingredients were respectively used as receptors and ligands. Binding energy can reflect the stability of protein receptor binding with small molecular ligands. The higher the absolute value of binding energy, the more stable the binding between receptor and ligand. The binding energy of −5.0 kcal/mol was set as a threshold for good binding between receptor and ligand. The ligand with the best binding to the receptor (the lowest energy) was selected according to the binding energy. The molecular docking results of the five groups of receptors with the lowest binding energy with ligands are shown in Figure 11 and Table 5.

**Table 5:** Molecular docking results of hub targets and key ingredients.

Hub targets (PDB ID)	Quercetin	Luteolin	Berberine	Isorhamnetin	Kaempferol
	(kcal/mol)	(kcal/mol)	(kcal/mol)	(kcal/mol)	(kcal/mol)
*ESR1(1ERE)*	−8.5	−7.9	−6.7	−7	−7.7
*JUN(1A02)*	−8.6	−8.7	−9.2	−8	−8.3
*SRC(1A07)*	−6.3	−7.3	−6.8	−6.7	−5.9
*PIK3R1(1A0N)*	−7.5	−7.3	−6.5	−6.9	−7.3
*FOS(1FOS)*	−8.3	−8.4	−8.4	−8.3	−8.1
*MAPK1(1PME)*	−6.6	−7.4	−6.5	−7.2	−7.4
*AKT1(7NH4)*	−8.2	−8.1	−7.5	−6.8	−7.6
*TP53(1A1U)*	−6.3	−6.4	−6.5	−6.1	−5.7
*RELA(INFI)*	−8.6	−9.1	−7.6	−8	−7.9
*MAPK3(6GES)*	−7.9	−8.3	−7.7	−7.7	−8

Quercetin and luteolin combined best with the hub targets. *JUN, FOS, AKT1, RELA,* and *MAPK3* interacted well with these critical ingredients. These indicated that some effective ingredients in the JPXK recipe had good binding properties with protein targets.

### 4.8. FBG, FINS, GC Results and HOMA‐IR Level

Compared with before treatment, after treatment, the blood sugar of the JPXK recipe group decreased, suggesting that the JPXK recipe had a good hypoglycemic effect. Compared with the control group, the FBG, HOMA‐IR, and GC values of the model group were significantly increased (*p* < 0.05), and the FINS value was decreased (*p* < 0.05). Compared with the model group, the FBG and GC values of the JPXK recipe group were significantly decreased (*p* < 0.05), and the FINS value was significantly increased (*p* < 0.05). The results are shown in Table 6. The concentration changes of FINS and GC in rats in each group are shown in Figures 12(a) and 12(b).

**Table 6:** Comparison of the levels of FBG, FINS, GC, and HOMA‐IR.

Group	*n*	FBG(mmol/L)		FINS(mIU/L)	GC(PG/L)	HOMA‐IR
		Before administration	after administration			
Control group	12	4.49 ± 0.16	4.67 ± 0.16	20.61 ± 0.54	152.78 ± 6.59	4.28 ± 0.16
Model group	12	26.38 ± 1.24^∗∗△^	26.96 ± 1.31^∗∗△^	12.61 ± 0.62^∗∗△^	228.75 ± 9.10^∗∗△^	15.12 ± 1.18^∗∗△^
JPXK recipe group	12	27.37 ± 1.38^∗∗△^	20.90 ± 1.13^∗∗△^	18.00 ± 0.39^∗∗△^	203.55 ± 6.82^∗∗△^	16.72 ± 0.93^∗∗△^

*Note:* Compared with the control group. FINS, Fasting serum insulin; GC, Serum glucagon; HOMA‐IR, Homeostatic Model Assessment for Insulin Resistance.

Abbreviation: FBG, fasting blood glucose.

^∗^ *p* < 0.05.

^∗∗^ *p* < 0.01; compared with the model group.

^△^ *p* < 0.05.

Figure 12Animal experiment results. (a) Fasting insulin value of each group. (b) Glucagon value of each group. (c–e) Immunofluorescence diagram of each group. Abbreviation: FINS, Fasting serum insulin; GC, Serum glucagon.

**Figure:**
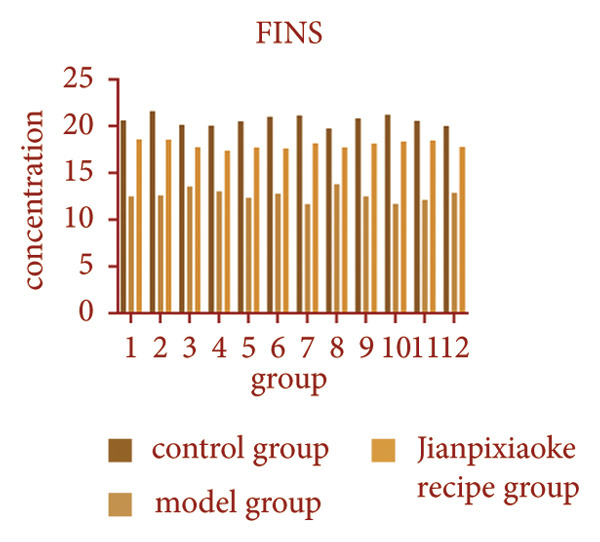
(a)

**Figure:**
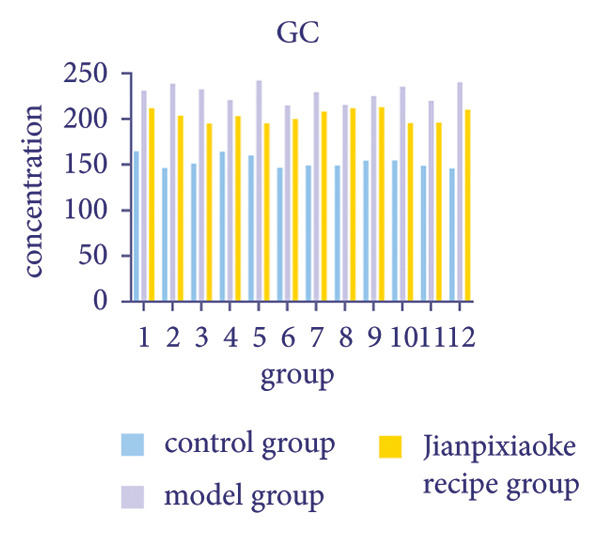
(b)

**Figure:**
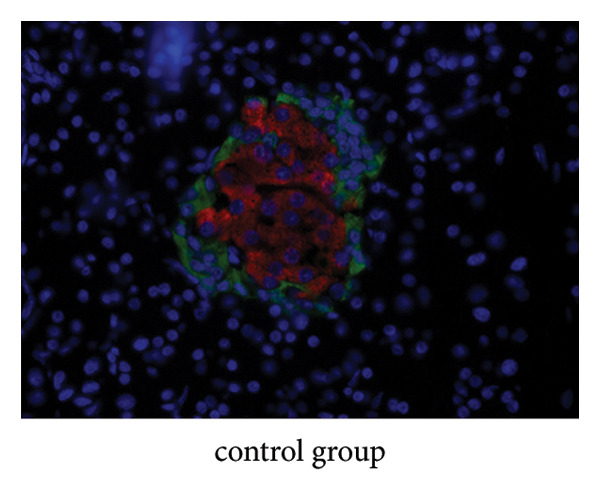
(c)

**Figure:**
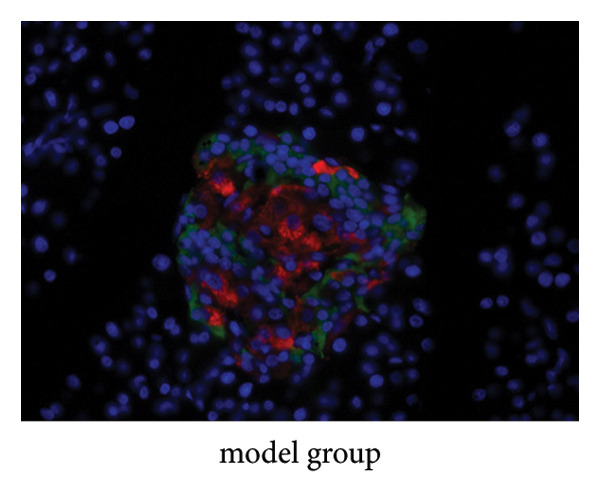
(d)

**Figure:**
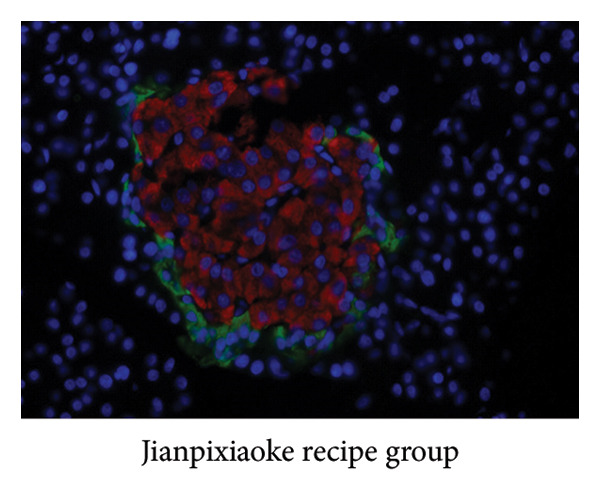
(e)

### 4.9. Immunofluorescence Observation of Pancreatic Tissue Distribution

Insulin was stained with red fluorescence in the cytoplasm, and glucagon was stained with green fluorescence. Immunofluorescence showed that the insulin in the JPXK recipe group was more distributed in the pancreas than the model group, which indicated that after the treatment of T2DM rats with the JPXK recipe, the islet function recovered and the level of insulin secretion was improved. The results are shown in Figures 12(c)–12(e).

## 5. Discussion

T2DM was a chronic metabolic disease characterized [19]. Long‐term high blood sugar leads to chronic damage and dysfunction of various tissues, especially the eyes, kidneys, blood vessels, and nerves. The JPXK recipe as a TCM compound significantly affected T2DM. Our team had confirmed through clinical and experimental studies that the JPXK recipe could lower blood sugar and improve pancreatic islet β cell function. Clinical studies had verified that the application of specific dosages of JPXK recipe could effectively reduce blood glucose, regulate blood lipid, improve IR, and preserve islet function in T2DM patients [20]; 689 patients had been treated with the JPXK recipe [21]. Experiments demonstrated that the application of specific dosages of JPXK recipe could significantly heighten the number of islet β cells in DM rats, inhibit the apoptosis of islet β cells [22], enhance autophagy of islet β cells, and promote insulin secretion, thus protecting islet function [23]. Concurrently, 7 therapeutic targets and biological processes of the application of specific dosages of JPXK recipe in the treatment of T2DM were found to adopt using microRNA omics technology, which was verified by experiments in later studies, enriching the research mechanism of JPXK recipe in the treatment of T2DM [24].

In our previous work, we applied network pharmacology to analyze the mechanism and potential pathways of HQ and HL (the main drugs in the JPXK recipe) and confirmed their effectiveness in the treatment of DM. HQ could ameliorate the insulin signaling pathway by upregulating casein kinase activity, regulating glucose and lipid metabolism, and enhancing IR, to treat T2DM [25]; HL could treat T2DM through an anti‐inflammatory effect [26]. At the same time, we conducted network pharmacology and molecular docking with the drug pair of HL and PL in the recipe for invigorating the spleen and eliminating thirst. The results showed that the drug pair could treat T2DM through anti‐inflammatory, regulating glucose and lipid metabolism, etc. [27]. These studies laid the foundation for the study of network pharmacology and molecular docking of JPXK recipe.

In this study, by constructing the PPI network of the JPXK recipe and T2DM intersection targets, we discovered that ESR1, JUN, SRC, PIK3R1, FOS, MAPK1, AKT1, TP53, RELA, and MAPK3 were the hub targets for the treatment of T2DM. These targets were mainly related to hosting immunity, glucose and lipid metabolism, and inflammatory response. The MAPK signaling pathway covered them, the cAMP signaling pathway, the tumor necrosis factor signaling pathway, and the Toll‐like receptor signaling pathway. We noted that only 6 hub targets overlap with 10 essential targets of T2DM. Based on the hub targets analysis, vital active ingredients and the main significant KEGG pathways explore the potential mechanism of the JPXK recipe in the treatment of T2DM.

### 5.1. Analysis of Hub Targets

The activation of the estrogen receptor gene (*ESR1*) could regulate hepatocyte factor, delay hepatic steatosis, and reduce IR for the sake of achieving the purpose of lowering blood glucose [28]. *AKT1* was a crucial protein factor of the phosphatidylinositol 3 kinase (PI3K)/AKT pathway [29], which could induce downstream molecules such as glucose transporters to improve glucose uptake, improve glucose utilization, participate in glucose metabolism and lipid metabolism, and regulate blood glucose balance [30]. It has been confirmed that *AKT1* could be expressed in many insulin‐sensitive tissues such as the liver, skeletal muscle, and fat [31]. *PIK3R1* was a member of the PI3K/AKT signaling pathway and participates in insulin metabolism. The increased expression of *PIK3R1* could promote the expression of pyruvate dehydrogenase kinase 4, inhibit glucose uptake and glycogen synthesis, delay insulin signal, and increase blood glucose in the body [32]. *JUN* was a signal molecule connecting inflammation and IR. *JUN* will be activated under long‐term hyperglycemia, promote the release of inflammatory factors, induce inflammation, enhance the expression of related apoptotic genes, and damage the function of islet cells [33]. Studies had shown that after the knockout of the *JUN* gene in DM mice, blood glucose was significantly reduced, insulin signal transduction was enhanced, and islet function was effectively improved [34]. As a tumor suppressor gene, *TP53* was related to body metabolism [35]. *TP53* could regulate glucose transporters on the cell membrane to enhance IR and blood glucose balance. Furthermore, it could affect glucose metabolism by intervening in glycolysis and other processes [36]. *SRC* was associated with a viral infection and tumors and played a role in activating the protein tyrosine kinase (PTK) family. Studies had shown that after *SRC* knockout in mice, under cold conditions, with the increase of heat production in mice, FBG level decreases and insulin sensitivity increases [37]. *FOS* was a proto‐oncogene that promoted apoptosis of pancreatic islet β cells, leading to a decrease in insulin levels in the body and the development of T2DM [38]. *MAPK1* and *MAPK3* belonged to a class of cytokines that could participate in inflammatory response and IR [39]. Studies had shown that *MAPK1* and *MAPK3* could enhance insulin secretion and regulate blood glucose stability [40]. *RELA* was an essential inflammatory factor, which could control the inflammatory response of cells, enhance the production of proinflammatory cytokines by macrophages, and aggravate the chronic inflammation and macrophage IR induced by a high‐fat diet [41]. Inhibition of *RELA* expression could increase glucose transport and consumption and improve macrophage IR [42].

### 5.2. Analysis of Vital Active Ingredients

Quercetin was a common ingredient of HQ, HL, and CNX. As a flavonoid, quercetin had antioxidant, anti‐inflammatory, hypoglycemic, and other pharmacological effects [43]. Research exhibited that quercetin could inhibit α‐glucosidase activity to promote glycogen synthesis and regulate the expression of *AKT1* and other related apoptotic genes to improve function of islet β cell [44], as well as activate *MAPK1* and *MAPK3*, increase insulin secretion, and improve IR, to regulate glucose metabolism [40]. Isorhamnetin had pharmacological effects such as antitumor, anti‐inflammatory, and antioxidant stress [45]. Isorhamnetin could regulate the Ras/MAPK signal pathway, lead to S‐phase arrest of PANC‐1 cells, and inhibit the proliferation of tumor cells [46]. Studies had shown that Isorhamnetin could hinder IKK/IKB/NF‐KB/iNOS signaling pathway and reduce interleukin‐1β(IL‐1β), interleukin‐6(IL‐6), and other inflammatory factors to achieve the effect of anti‐DM [47]. Luteolin had anti‐inflammatory, antioxidant, and other pharmacological effects, which could inhibit inflammatory factors such as interleukin‐1 (IL‐1) and IL‐6, promote the expression of anti‐inflammatory factors, reduce the inflammatory response, and improve IR [48, 49]. TNF signaling pathway was one of the critical pathways of inflammatory response in the body and participates in the occurrence and development of T2DM [50]. Studies had shown that luteolin could inhibit the activation of NF‐Kb in intracellularization, reducing the release of TNF‐α, which was a therapeutic effect of DM [51]. Berberine had physiological functions such as antibacterial, hypoglycemic, and lipid‐lowering, which could promote the process of islet β cell regeneration, effectively reduce IR, and could reduce the activity of maltase and sugars to reduce the postprandial blood sugar of patients with T2DM [52]. Studies had shown that berberine could heighten ghrelin and GLP‐1 in gastrointestinal hormones, increase the role of gastrointestinal hormones, and regulate glucose and lipid metabolism [52]. Berberine also could restrict the insulin gene Ins2 promoter, activate amp‐activated protein kinase (AMPK) in the liver and muscle, uphold mitochondrial uncoupling protein 2 (UCP2), increase energy consumption, and promote glucose metabolism [53]. Kaempferol had antioxidant, anti‐inflammatory, hypoglycemic, anticancer, and other pharmacological effects [54]. Kaempferol could boost glucose metabolism in skeletal muscle, inhibit liver gluconeogenesis, increase sensitivity to insulin, and promote insulin secretion to achieve the effect of treating T2DM [55]. Kaempferol also could likewise protect the islet β cells by reducing the activity of T cells and regulating Th1/Th2 [56]. Research on Fang Min and others showed that kaempferol could adjust TNF‐α, IL‐17, AGE‐RAGE, and other signal channels through critical targets such as AKT1 and TNF‐α to treat T2DM [57].

### 5.3. Analysis of Critical KEGG Pathways

Toll‐like receptor signaling pathway played an essential role in inflammatory response and IR in patients with DM and could regulate immune response and inflammatory response [58]. The cyclic adenosine monophosphate (CAMP)/protein kinase (PKA) signaling pathway could regulate glucose homeostasis in various ways, including glucose uptake, glycogen synthesis, and decomposition [59]. The MAPK signaling pathway involved chronic DM complications and was associated with high glucose, oxidative stress, growth factors, and other related in DM patients. Activating MAPK signaling pathway could inhibit IRS phosphorylation and increase IR [60]. As a vital path of inflammation of the body, the TNF signaling pathway was closely related to the NF‐KB signaling pathway and jointly participates in the development process of T2DM [61]. TNF‐α activates the TNF signaling pathway by combining the receptor TNFR1, raises downstream signal protein molecules, maintains the continuous activation of NF‐kB, forms an inflammatory response, and aggravates IR [62]. Thyroid hormone improves the sensitivity of the body to insulin by regulating hepatic glycogen synthesis and increasing glucose utilization, to regulate the stability of blood glucose in the body [63]. The role of the estrogen signaling pathway was mainly related to estrogen receptor (ER) mediated [64]. Studies had shown that ER could regulate PIK3/Akt signaling pathway, promote Nrf2 signal transduction, increase antioxidant enzyme levels, and inhibit oxidative damage to DM complications [65]. Human giant cell virus infections had caused damage to the body’s immune mechanism, and a large amount of islet β cells have apoptosis, which had promoted the rise of blood sugar, which had caused T2DM for a long time [66]. The infection of human immunodeficiency virus type 1 infection destroys the immune system, aggravating DM prevalence [67]. T2DM was closely related to COVID‐19. Infection of COVID‐19 by the body could lead to a massive release of proinflammatory cytokines, resulting in the chronic inflammatory reaction, and decreasing insulin secretion, thereby aggravating the risk of DM [68]. For people without DM, it might likewise increase the risk of hyperglycemia and increase the risk of critical illness [69].

The results demonstrated that the essential targets and KEGG pathways of the T2DM–JPXK recipe were all related to cancer. Research had also shown that DM was associated with numerous cancers, such as breast cancer and colorectal cancer [70], whereas the molecular mechanism of the two was unclear, which might be due to the long‐term hyperglycemia of T2DM patients, which provided nutrition for the growth of tumor cells, improved the expression of insulin‐like growth factor (IGF) and receptors in the state of IR and hyperinsulinemia, and promoted the proliferation of tumor cells, thus increasing the risk of T2DM patients suffering from tumor, which also corresponds to the KEGG pathway screened in this paper [71, 72]. It might also be the metabolic disorder of T2DM patients, increasing reactive oxygen species, and long‐term oxidative damage of DNA causes cancer [70]. At present, metformin, the first‐line drug of T2DM, had an anticancer effect [73]. In patients with hepatitis B, due to liver damage in vivo, the synthesis of liver glycogen was reduced, and the level of glucose in vivo was increased. In the case of long‐term hyperglycemia, pancreatic islet cells will work in compensation, which will lead to IR, insufficient insulin secretion, and ultimately lead of DM [74]. Zhang Huiyan considered that chronic infection of the hepatitis B virus was closely related to liver‐derived DM [75].

In summary, in this study, network pharmacology and molecular docking technology were used to preliminarily explore the complex mechanism of JPXK Recipe in treating T2DM through multicomponent, multitarget, and multichannel. The consequences demonstrated that the components of the JPXK recipe directly affected DM and might play a critical role in DM by regulating the metabolism of glucose and lipid, participating in the process of lipopolysaccharide reaction, IR, and related signal transduction. Simultaneously, the relationship between DM and cancer showed that the JPXK recipe might not only treat T2DM but also effectively prevent the occurrence of cancer. Molecular docking revealed that quercetin and luteolin combined best with the hub targets. *JUN*, *FOS*, *AKT1*, *RELA*, and *MAPK3* interacted well with these critical ingredients. Animal experiments had verified that the JPXK recipe could effectively treat T2DM by lowering blood sugar and protecting islet function. Notwithstanding, this study only confirmed the participation of the JPXK recipe in hypoglycemic, anti‐inflammatory, and other mechanisms from a microperspective. Subsequent examinations will be experimentally and clinically verified to make them more credible and clinically significant.

## 6. Conclusion

The key active ingredients of the JPXK recipe for the treatment of T2DM included quercetin [76, 77], isorhamnetin, luteolin, berberine, and kaempferol. Through the construction of the compound targets disease network, the 10 hub targets including *ESR1*, *JUN*, *SRC*, *PIK3R1*, *FOS*, *MAPK1*, *AKT1*, *TP53*, *RELA*, and *MAPK3* were obtained. The main pathways of the JPXK recipe in treating T2DM included the MAPK signaling pathway, cAMP signaling pathway, tumor necrosis factor signaling pathway, Toll‐like receptor signaling pathway, etc. Animal experiments demonstrated that the JPXK recipe had a great hypoglycemic effect and could effectively protect the function of pancreatic islets. The therapeutic effect of the JPXK recipe in treating T2DM was mainly through regulating the host’s immune and inflammatory response, antibacterial microorganisms, regulating glucose and lipid metabolism, improving IR, etc. This study could provide a theoretical basis for further exploration of the treatment of T2DM with JPXK recipe. However, this study only explored the mechanism of action of specific TCM formulas in treating T2DM, and the theoretical basis provided in the exploration of TCM in treating T2DM had certain limitations.

## Disclosure

All authors approved the submitted version.

## Conflicts of Interest

The authors declare no conflicts of interest.

## Author Contributions

Y.H. and H.L. conceived and designed this study. Y.H., H.L., and W.A. conducted data analysis and data interpretation. C.H., G.F., and H.L. conducted animal experiments and data analysis. Y.H. and H.L. conducted the statistical analysis and prepared the manuscript. All authors contributed to the article.

## Funding

This research was supported by the National Natural Science Foundation of China (81974562), Shandong Taishan Scholar Project (tsqn202211354), Qiuhai Qian national famous traditional Chinese Medicine expert inheritance studio (National Traditional Chinese Medicine Education Letter [2022] No. 75), Shandong Key Laboratory of Traditional Chinese Medicine Efficacy and Mechanism (PKL2024C23), and Construction and Efficacy Evaluation of an Intelligent Innovation Platform for Weight Management utilizing Traditional Chinese Medicine (2025CXPT147).

## Data Availability

The data used to support the findings of this study are available from the corresponding author upon request.

## References

[1] Porte D.Jr, Clinical Importance of Insulin Secretion and Its Interaction with Insulin Resistance in the Treatment of Type 2 Diabetes Mellitus and Its Complications, Diabetes/metabolism research and reviews. (2001) 17, no. 3, 181–188, 10.1002/1520-7560(200105/06)17:3<181::aid-dmrr197>3.0.co;2-1. 11424231

[2] Sun H. , Saeedi P. , Karuranga S. et al., IDF Diabetes Atlas: Global, Regional and Country-Level Diabetes Prevalence Estimates for 2021 and Projections for 2045, Diabetes Research and Clinical Practice. (2022) 183, 10.1016/j.diabres.2021.109119. PMC11057359 34879977

[3] Dou Z. , Xia Y. , Zhang J. et al., Syndrome Differentiation and Treatment Regularity in Traditional Chinese Medicine for Type 2 Diabetes: A Text Mining Analysis, Frontiers in Endocrinology. (2021) 12, 10.3389/fendo.2021.728032. PMC8733618 35002950

[4] Ramírez-Alarcón K. , Victoriano M. , Mardones L. et al., Phytochemicals as Potential Epidrugs in Type 2 Diabetes Mellitus, Frontiers in Endocrinology. (2021) 12, 10.3389/fendo.2021.656978. PMC8204854 34140928

[5] Jie H. , Yunsheng X. , and Huang Y. , ‘Based on the Theory Of Spleen Deficiency Causing Diabetes’, the Research Assumption on the Mechanism of Invigorating the Spleen and Clearing Heat to Prevent and Treat Diabetes Through AMPK/mTORC1/SAD-A Pathway, Chinese Journal of Traditional Chinese Medicine. (2019) 34, no. 03, 1104–1107, in Chinese.

[6] Luo D. , Huang J. , Fang G. , Dong X. , and Huang Y. , Research on the Effect of Jianpixiaoke Recipe Based on the Intestinal Pancreatic Axis On the Function of Pancreatic Islet β Cells in Patients with Metabolic Syndrome, New Chinese Medicine and Clinical Pharmacology. (2022) 33, no. 08, 1118–1123, in Chinese.

[7] Yanqin H. , Jie H. , Wenrong An , Luo D. , and Yunsheng Xu , Based on cTAGE5/TANGO1 Intracellular Transport, Jianpixiaoke Recipe Improves Endoplasmic Reticulum Stress to Protect Pancreatic Islets β Cellular Mechanisms, Chinese Journal of Gerontology. (2023) 43, no. 12, 3051–3054, in Chinese.

[8] Zhou Z. , Chen B. , Chen S. et al., Applications of Network Pharmacology in Traditional Chinese Medicine Research, Evidence-Based Complementary and Alternative Medicine. (2020) 2020, no. 1, 10.1155/2020/1646905. PMC7042531 32148533

[9] Zhang R. , Zhu X. , Bai H. , and Ning K. , Network Pharmacology Databases for Traditional Chinese Medicine: Review and Assessment, Frontiers in Pharmacology. (2019) 10, 10.3389/fphar.2019.00123, 2-s2.0-85065698881. PMC6393382 30846939

[10] Sivakumar K. C. , Haixiao J. , Naman C. B. , and Sajeevan T. P. , Prospects of Multitarget Drug Designing Strategies by Linking Molecular Docking and Molecular Dynamics to Explore the protein-ligand Recognition Process, Drug Development Research. (2020) 81, no. 6, 685–699, 10.1002/ddr.21673. 32329098

[11] Gfeller D. , Grosdidier A. , Wirth M. , Daina A. , Michielin O. , and Zoete V. , Swisstargetprediction: A Web Server for Target Prediction of Bioactive Small Molecules, Nucleic Acids Research. (2014) 42, no. W1, W32–W38, 10.1093/nar/gku293, 2-s2.0-84904793576. 24792161 PMC4086140

[12] Kuhn M. , Szklarczyk D. , Franceschini A. et al., STITCH 2: An Interaction Network Database for Small Molecules and Proteins, Nucleic Acids Research. (2010) 38, no. suppl_1, D552–D556, 10.1093/nar/gkp937, 2-s2.0-75549087827. 19897548 PMC2808890

[13] Meng Z. , Liu X. , Wu J. et al., Mechanisms of Compound Kushen Injection for the Treatment of Lung Cancer Based on Network Pharmacology, Evidence-Based Complementary and Alternative Medicine. (2019) 2019, 1–15, 10.1155/2019/4637839, 2-s2.0-85067100629. PMC6558614 31275410

[14] He J. , Yuan G. , Zhang J. , and Guo X. , Establishment of Rat Model of Early Diabetes Peripheral Neuropathy, Journal of Peking University. (2019) 51, no. 06, 1150–1154, in Chinese. 10.19723/j.issn.1671-167X.2019.06.030 PMC7433606 31848520

[15] Xie S. , Huang L. , Hongjie D. , and Chao L. , Research Progress in the Prevention and Treatment of Obesity Based on the Regulation of Gut Microbiota in Traditional Chinese Medicine, World Science and Technology Modernization of Traditional Chinese Medicine. (2019) 21, no. 11, 2474–2479, in Chinese.

[16] Karlsson F. H. , Tremaroli V. , Nookaew I. et al., Gut Metagenome in European Women with Normal, Impaired and Diabetic Glucose Control, Nature. (2013) 498, no. 7452, 99–103, 10.1038/nature12198, 2-s2.0-84878709716. 23719380

[17] Tai N. , Wong F. S. , and Wen L. , The Role of Gut Microbiota in the Development of Type 1, Type 2 Diabetes Mellitus and Obesity, Reviews in Endocrine & Metabolic Disorders. (2015) 16, no. 1, 55–65, 10.1007/s11154-015-9309-0, 2-s2.0-84925534569. 25619480 PMC4348024

[18] Ou L. , Li X. , Li Yu , Li J. , and Long F. , Clinical Observation on the Treatment of Phlegm (Dampness) and Heat Syndrome of Newly Diagnosed Type 2 Diabetes with Modified Huanglian Wendan Decoction, Chinese Journal of Experimental Prescriptions. (2021) 27, no. 01, 128–134, in Chinese.

[19] Nair A. T. N. , Wesolowska-Andersen A. , Brorsson C. et al., Heterogeneity in Phenotype, Disease Progression and Drug Response in Type 2 Diabetes, Nature Medicine. (2022) 28, no. 5, 982–988, 10.1038/s41591-022-01790-7. 35534565

[20] Wenrong A. , Clinical Observation of Jianpixiaoke Recipe in the Treatment of T2DM and Experimental Study on Regulating Islet β Cell Function Through AMPK/mTOR Pathway, Master’s degree. (2019) in Chinese.

[21] Wen R. A. , Xu Y. S. , and Huang Y. Q. , Analysis of Clinical Characteristics and Rules of Jianpixiaoke Recipe for Type 2 Diabetes Based on Data Mining, Chinese Journal of Traditional Chinese Medicine. (2020) 38, no. 02, 191–194, in Chinese.

[22] Yanqin H. , Effect of Jianpixiaoke Recipe on the Secretory Function of Pancreatic Islet Cells in Type 2 Diabetes Rats, Journal of Traditional Chinese Medicine. (2015) 56, no. 03, 249–252, in Chinese.

[23] Zhou J. , Xu Y. , Li J. , Xu C. , and Huang Y. , The Mechanism of Jianpixiaoke Recipe Regulating Autophagy to Improve Pancreatic Islet Function in Type 2 Diabetes Rats, Chinese Journal of Traditional Chinese Medicine. (2021) 36, no. 08, 4556–4560, in Chinese.

[24] Guo Q. , Xu Y. , Li J. et al., Probe into the Target and Mechanism of Jianpi Xiaoke Prescription for Treating Type 2 Diabetes Mellitus Through Mirna Expression Profiling, Evidence-based Complementary and Alternative Medicine. (2020) 2020, no. 1, 10.1155/2020/7370350. PMC7785360 33456489

[25] Li J. , Huang Y. , Zhao S. et al., Based on Network Pharmacology to Explore the Molecular Mechanisms of Astragalus membranaceus for Treating T2 Diabetes Mellitus, Annals of Translational Medicine. (2019) 7, no. 22, 10.21037/atm.2019.10.118. PMC6944577 31930034

[26] An W. , Huang Y. , Chen S. et al., Mechanisms of Rhizoma Coptidis Against Type 2 Diabetes Mellitus Explored by Network Pharmacology Combined with Molecular Docking and Experimental Validation, Scientific Reports. (2021) 11, no. 1, 10.1038/s41598-021-00293-8. PMC8531350 34675276

[27] Li H. , Luo D. , Wei R. et al., Investigating the Mechanism of Rhizoma Coptidis-Eupatorium Fortunei Medicine in the Treatment of Type 2 Diabetes Based on Network Pharmacology and Molecular Docking, BioMed Research International. (2022) 2022, no. 1, 10.1155/2022/7978258. PMC9705109 36452059

[28] Zhang Y. Y. , Li C. , Yao G. F. et al., Deletion of Macrophage Mineralocorticoid Receptor Protects Hepatic Steatosis and Insulin Resistance Through ERα/HGF/Met Pathway, Diabetes. (2017) 66, no. 6, 1535–1547, 10.2337/db16-1354, 2-s2.0-85019644236. 28325853 PMC5860190

[29] Zhang Z. , Liu H. , and Liu J. , Akt Activation: A Potential Strategy to Ameliorate Insulin Resistance, Diabetes Research and Clinical Practice. (2019) 156, 10.1016/j.diabres.2017.10.004, 2-s2.0-85056279427. 29111280

[30] Alwhaibi A. , Verma A. , Adil M. S. , and Somanath P. R. , The Unconventional Role of Akt1 in the Advanced Cancers and in diabetes-promoted Carcinogenesis, Pharmacological Research. (2019) 145, 10.1016/j.phrs.2019.104270, 2-s2.0-85065656567. PMC6659399 31078742

[31] Ou R. , Mo L. , Tang H. et al., circRNA-AKT1 Sequesters miR-942-5p to Upregulate AKT1 and Promote Cervical Cancer Progression, Molecular Therapy-Nucleic Acids. (2020) 20, 308–322, 10.1016/j.omtn.2020.01.003. 32193155 PMC7078494

[32] Zheng Y. , Lang Y. , Qi Z. , Gao W. , Hu X. , and Li T. , PIK3R1, SPNB2, and CRYAB as Potential Biomarkers for Patients with Diabetes and Developing Acute Myocardial Infarction, International journal of endocrinology. (2021) 2021, 1–15, 10.1155/2021/2267736. PMC8651423 34887920

[33] Wang Q. , Zhang H. , Zhao B. , and Fei H. , IL-1β Caused Pancreatic β-cells Apoptosis Is Mediated in Part by Endoplasmic Reticulum Stress via the Induction of Endoplasmic Reticulum Ca2+ Release Through the c-Jun N-terminal Kinase Pathway, Molecular and Cellular Biochemistry. (2009) 324, no. 1-2, 183–190, 10.1007/s11010-008-9997-9, 2-s2.0-61549086548. 19109696

[34] Yang J. , Rong Y. , Wu Y. , Liu X. , and Yihui D. , Exploring the Molecular Mechanism of Shenqi Pill Intervention in Type 2 Diabetes Based on Network Pharmacology and Verifying Its Key Action Pathway, Journal of Beijing University of Chinese Medicine. (2021) 44, no. 01, 60–68, in Chinese.

[35] Sliwinska A. , Kasznicki J. , Kosmalski M. et al., Tumour Protein 53 Is Linked with Type 2 Diabetes Mellitus, Indian Journal of Medical Research. (2017) 146, no. 2, 237–243, 10.4103/ijmr.IJMR_1401_15, 2-s2.0-85040511846. 29265025 PMC5761034

[36] Kung C. P. and Murphy M. E. , The Role of the p53 Tumor Suppressor in Metabolism and Diabetes, Journal of Endocrinology. (2016) 231, no. 2, R61–R75, 10.1530/JOE-16-0324, 2-s2.0-85006001577. 27613337 PMC5148674

[37] Visscher T. L. , Heitmann B. L. , Rissanen A. , Lahti-Koski M. , and Lissner L. , A Break in the Obesity Epidemic? Explained by Biases or Misinterpretation of the Data?, International Journal of Obesity. (2015) 39, no. 2, 189–198, 10.1038/ijo.2014.98, 2-s2.0-84922660421. 24909829

[38] Abdel-Megeed R. M. , El Newary S. A. , Kadry M. O. et al., Hyssopus Officinalis Exerts Hypoglycemic Effects on Streptozotocin-Induced Diabetic Rats via Modulating GSK-3β, C-fos, NF-κB, ABCA1 and ABGA1 Gene Expression, Journal of Diabetes and Metabolic Disorders. (2020) 19, no. 1, 483–491, 10.1007/s40200-020-00535-y. 32550200 PMC7271080

[39] Cui X. , Qian D. W. , Jiang S. , Shang E. X. , Zhu Z. H. , and Duan J. A. , Scutellariae Radix and Coptidis Rhizoma Improve Glucose and Lipid Metabolism in T2DM Rats via Regulation of the Metabolic Profiling and MAPK/PI3K/Akt Signaling Pathway, International Journal of Molecular Sciences. (2018) 19, no. 11, 10.3390/ijms19113634, 2-s2.0-85056727916. PMC6274950 30453687

[40] Yan S. , Xian L. , Sun C. , and Chen K. , Research Progress on the Hypoglycemic and Lipid-Lowering Activity of Quercetin and Its Glycoside Derivatives, Chinese Journal of Traditional Chinese Medicine. (2015) 40, no. 23, 4560–4567, in Chinese. 27141664

[41] Mukherjee N. , Houston T. J. , Cardenas E. , and Ghosh R. , To Be an Ally or an Adversary in Bladder Cancer: the NF-κB Story has Not Unfolded, Carcinogenesis. (2015) 36, no. 3, 299–306, 10.1093/carcin/bgu321, 2-s2.0-84924503690. 25543121 PMC4425835

[42] Kang X. , Hou A. , Wang R. et al., Macrophage TCF-4 co-activates p65 to Potentiate Chronic Inflammation and Insulin Resistance in Mice, Clinical Science (London, England: 1979). (2016) 130, no. 14, 1257–1268, 10.1042/CS20160192, 2-s2.0-85014100086. 27129186

[43] Yi H. , Peng H. , Wu X. et al., The Therapeutic Effects and Mechanisms of Quercetin on Metabolic Diseases: Pharmacological Data and Clinical Evidence, Oxidative Medicine and Cellular Longevity. (2021) 2021, no. 1, 10.1155/2021/6678662. PMC8249127 34257817

[44] Feng Y. , Li H. , Liu J. , Ruan Z. , and Zhai G. , Research Progress of Quercetin, Chinese Journal of Traditional Chinese Medicine. (2021) 46, no. 20, 5185–5193, in Chinese 10.19540/j.cnki.cjcmm.20210524.602. 34738418

[45] Gong G. , Guan Y. Y. , Zhang Z. L. et al., Isorhamnetin: A Review of Pharmacological Effects, Biomedicine & Pharmacotherapy. (2020) 128, 10.1016/j.biopha.2020.110301. 32502837

[46] Zhao F. , Hou L. , Yang Y. et al., Research Progress on the Anti-Tumor Mechanism of Isorhamnetin, China Pharmacy. (2021) 32, no. 24, 3054–3059, in Chinese.

[47] Lai Q. H. , Xiong Q. , Wu G. , He G. Z. , and Xu Y. C. , Study on the Protective Effect of Isorhamnetin on Islet β Cell Injury Induced by High Glucose and High-Fat, Chinese Journal of diabetes. (2020) 28, no. 06, 461–466, in Chinese.

[48] Qian Y. and Guanzhong W. , Research Progress on Anti-inflammatory Mechanism of Luteolin, Pharmaceutical Research. (2019) 38, no. 02, 108–111, in Chinese.

[49] Wang Q. , Li K. , and Zhou C. , Research Progress on Pharmacological Effects and Preparations of Luteolin, Journal of Beijing Union University. (2022) 36, no. 01, 59–63, in Chinese.

[50] Niewczas M. A. , Gohda T. , Skupien J. et al., Circulating TNF Receptors 1 and 2 Predict ESRD in Type 2 Diabetes, Journal of the American Society of Nephrology. (2012) 23, no. 3, 507–515, 10.1681/ASN.2011060627, 2-s2.0-84857938428. 22266663 PMC3294310

[51] Kim H. J. , Lee W. , and Yun J. M. , Luteolin Inhibits Hyperglycemia-Induced Proinflammatory Cytokine Production and Its Epigenetic Mechanism in Human Monocytes, Phytotherapy Research. (2014) 28, no. 9, 1383–1391, 10.1002/ptr.5141, 2-s2.0-84907927150. 24623679

[52] Chen Z. , Wang C. , Xu L. , and Liu Y. , The Effect of Ber-berine on Gastrointestinal Hormones and Glucose and Lipid Metabolism in Patients with Early Type 2 Diabetes, Contemporary Chinese Medicine. (2021) 28, no. 34, 120–122, in Chinese.

[53] Shen N. , Huan Y. , and Shen Z. F. , Berberine Inhibits Mouse Insulin Gene Promoter Through Activation of AMP Activated Protein Kinase and may Exert Beneficial Effect on Pancreatic β-cell, European Journal of Pharmacology. (2012) 694, no. 1-3, 120–126, 10.1016/j.ejphar.2012.07.052, 2-s2.0-84867337756. 22955013

[54] Wang J. , Mao J. , Wang R. , Li S. , Wu B. , and Yuan Y. , Kaempferol Protects Against Cerebral Ischemia Reperfusion Injury Through Intervening Oxidative and Inflammatory Stress Induced Apoptosis, Frontiers in Pharmacology. (2020) 11, 10.3389/fphar.2020.00424. PMC7174640 32351385

[55] Bai L. , Li X. , He L. et al., Antidiabetic Potential of Flavonoids from Traditional Chinese Medicine: A Review, The American Journal of Chinese Medicine. (2019) 47, no. 05, 933–957, 10.1142/S0192415X19500496, 2-s2.0-85068521743. 31248265

[56] Liu G. , Liu Y. , Cheng S. , Bao H. , and Yu J. , Effects of Kaempferol on Glucose and Lipid Metabolism and Insulin Resistance in Type 2 Diabetes Rats, Journal of Practical Clinical Medicine. (2012) 16, no. 09, 1–3, in Chinese.

[57] Wang S. , Huang H. , Su C. , Fan L. , Huang M. , and Fang M. , Research on the Mechanism of Kaempferol in the Treatment of Type 2 Diabetes with Xuefu Zhuyu Decoction Based on Network Pharmacology, Modern Chinese Doctors. (2021) 59, no. 22, 17–19, in Chinese.

[58] Wu J. , Lu Y. , and Liu C. , Toll like Receptors and Diabetes, International Journal of Endocrinology and Metabolism. (2008) 28, no. 2, 116–118, in Chinese.

[59] Yang H. and Yang L. , Targeting cAMP/PKA Pathway for Glycemic Control and Type 2 Diabetes Therapy, Journal of Molecular Endocrinology. (2016) 57, no. 2, R93–R108, 10.1530/JME-15-0316, 2-s2.0-84978924508. 27194812

[60] Choi J. , Kim K. J. , Koh E. J. , and Lee B. Y. , Gelidium Elegans Extract Ameliorates Type 2 Diabetes via Regulation of MAPK and PI3K/Akt Signaling, Nutrients. (2018) 10, no. 1, 10.3390/nu10010051, 2-s2.0-85040545728. PMC5793279 29316644

[61] Akash M. S. H. , Rehman K. , and Liaqat A. , Tumor Necrosis Factor-Alpha: Role in Development of Insulin Resistance and Pathogenesis of Type 2 Diabetes Mellitus, Journal of Cellular Biochemistry. (2018) 119, no. 1, 105–110, 10.1002/jcb.26174, 2-s2.0-85021199618. 28569437

[62] Varfolomeev E. , Goncharov T. , Maecker H. et al., Cellular Inhibitors of Apoptosis are Global Regulators of NF-κB and MAPK Activation by Members of the TNF Family of Receptors, Science Signaling. (2012) 5, no. 216, 10.1126/scisignal.2001878, 2-s2.0-84858627919. 22434933

[63] Hu D. and Jia X. , Change of Serum Thyroid Hormones Level in Patients with Type 2 Diabetes and its Clinical Significance, Immunoassay and clinical. (2015) 22, no. 5, 405–407, in Chinese.

[64] Zhu C. , Wang S. , Wang B. et al., 17β-Estradiol up-regulates Nrf2 via PI3K/AKT and Estrogen Receptor Signaling Pathways to Suppress light-induced Degeneration in Rat Retina, Neuroscience. (2015) 304, 328–339, 10.1016/j.neuroscience.2015.07.057, 2-s2.0-84938903109. 26211446

[65] Wu P. , Yan Y. , Ma L. L. et al., Effects of the Nrf2 Protein Modulator Salvianolic Acid A Alone or Combined with Metformin on diabetes-associated Macrovascular and Renal Injury, Journal of Biological Chemistry. (2016) 291, no. 42, 22288–22301, 10.1074/jbc.M115.712703, 2-s2.0-84988028606. 27417135 PMC5064007

[66] Wang L. M. , Chen W. F. , Liu J. B. , Wang S. , and Yuan Q. , Study on the Effect of HCMV Infection on Glucose Metabolism in Type 2 Diabetes, Journal of Infectiology of China Hospital. (2019) 29, no. 10, 1500–1503, in Chinese.

[67] Calza L. , Masetti G. , Piergentili B. et al., Prevalence of Diabetes Mellitus, Hyperinsulinaemia and Metabolic Syndrome Among 755 Adult Patients with HIV-1 Infection, International Journal of STD & AIDS. (2011) 22, no. 1, 43–45, 10.1258/ijsa.2010.010256, 2-s2.0-79952257206. 21364066

[68] Zhou Y. , Chi J. , Lv W. , and Wang Y. , Obesity and Diabetes as High-Risk Factors for Severe Coronavirus Disease 2019 (Covid-19), Diabetes/Metabolism Research and Reviews. (2021) 37, no. 2, 10.1002/dmrr.3377. PMC7361201 32588943

[69] Feldman E. L. , Savelieff M. G. , Hayek S. S. , Pennathur S. , Kretzler M. , and Pop-Busui R. , COVID-19 and Diabetes: A Collision and Collusion of Two Diseases, Diabetes. (2020) 69, no. 12, 2549–2565, 10.2337/dbi20-0032. 32938731 PMC7679769

[70] Srivastava S. P. and Goodwin J. E. , Cancer Biology and Prevention in Diabetes, Cells. (2020) 9, no. 6, 10.3390/cells9061380. PMC7349292 32498358

[71] Zhang Y. , Minglong L. , and Chen H. , Research Progress on the Relation-Ship Between Type 2 Diabetes and Hypoglycemic Drugs and Cancer, Shandong Medi-cine. (2019) 59, no. 33, 109–111, in Chinese.

[72] Zhu B. and Qu S. , The Relationship Between Diabetes Mellitus and Cancers and its Underlying Mechanisms, Frontiers in Endocrinology. (2022) 13, 10.3389/fendo.2022.800995. PMC8873103 35222270

[73] Landman G. W. , Kleefstra N. , van Hateren K. J. , Groenier K. H. , Gans R. O. , and Bilo H. J. , Metformin Associated with Lower Cancer Mortality in Type 2 Diabetes: ZODIAC-16, Diabetes Care. (2010) 33, no. 2, 322–326, 10.2337/dc09-1380, 2-s2.0-75149179169. 19918015 PMC2809274

[74] Wu R. , Shi X. , Xu J. , Zhong S. , and Lin X. , Analysis of the Relationship Between Diabetes and Hepatitis B Virus Infection, Journal of Math-Ematical Medicine. (2019) 32, no. 1, 48–49, in Chinese.

[75] Zhang H. , Investigation on the Prevalence of Type 2 Diabetes in Patients with Chronic Hepatitis B and Analysis of Related Factors, China’s health industry. (2014) no. 33, 56–57, in Chinese.

[76] Basaldúa-Maciel V. , Guzmán-Flores J. M. , Reyes-Chaparro A. , and Martínez-Esquivias F. , Therapeutic Potential of Quercetin in Type 2 Diabetes Based on a Network Pharmacology Study, Current Topics in Medicinal Chemistry. (2025) 10.2174/0115680266361598250212030220. 39949091

[77] Martínez-Esquivias F. , Guzmán-Flores J. M. , Pech-Santiago E. O. , Guerrero-Barrera A. L. , Delgadillo-Aguirre C. K. , and Anaya-Esparza L. M. , Therapeutic Role of Quercetin in Prostate Cancer: A Study of Network Pharmacology, Molecular Docking, and Dynamics Simulation, Cell Biochemistry and Biophysics. (2025) 10.1007/s12013-025-01697-3. 39966335

